# Access to and safety of COVID-19 convalescent plasma in the United States Expanded Access Program: A national registry study

**DOI:** 10.1371/journal.pmed.1003872

**Published:** 2021-12-20

**Authors:** Jonathon W. Senefeld, Patrick W. Johnson, Katie L. Kunze, Evan M. Bloch, Noud van Helmond, Michael A. Golafshar, Stephen A. Klassen, Allan M. Klompas, Matthew A. Sexton, Juan C. Diaz Soto, Brenda J. Grossman, Aaron A. R. Tobian, Ruchika Goel, Chad C. Wiggins, Katelyn A. Bruno, Camille M. van Buskirk, James R. Stubbs, Jeffrey L. Winters, Arturo Casadevall, Nigel S. Paneth, Beth H. Shaz, Molly M. Petersen, Bruce S. Sachais, Matthew R. Buras, Mikolaj A. Wieczorek, Benjamin Russoniello, Larry J. Dumont, Sarah E. Baker, Ralph R. Vassallo, John R. A. Shepherd, Pampee P. Young, Nicole C. Verdun, Peter Marks, N. Rebecca Haley, Robert F. Rea, Louis Katz, Vitaly Herasevich, Dan A. Waxman, Emily R. Whelan, Aviv Bergman, Andrew J. Clayburn, Mary Kathryn Grabowski, Kathryn F. Larson, Juan G. Ripoll, Kylie J. Andersen, Matthew N. P. Vogt, Joshua J. Dennis, Riley J. Regimbal, Philippe R. Bauer, Janis E. Blair, Zachary A. Buchholtz, Michaela C. Pletsch, Katherine Wright, Joel T. Greenshields, Michael J. Joyner, R. Scott Wright, Rickey E. Carter, DeLisa Fairweather

**Affiliations:** 1 Department of Anesthesiology and Perioperative Medicine, Mayo Clinic, Rochester, Minnesota, United States of America; 2 Department of Quantitative Health Sciences, Mayo Clinic, Jacksonville, Florida, United States of America; 3 Department of Quantitative Health Sciences, Mayo Clinic, Scottsdale, Arizona, United States of America; 4 Division of Transfusion Medicine, Department of Pathology, Johns Hopkins University, Baltimore, Maryland, United States of America; 5 Department of Anesthesiology, Cooper Medical School of Rowan University, Cooper University Health Care, Camden, New Jersey, United States of America; 6 Department of Pathology and Immunology, Washington University School of Medicine in St. Louis, St. Louis, Missouri, United States of America; 7 ImpactLife, Davenport, Iowa, United States of America; 8 Department of Cardiovascular Medicine, Mayo Clinic, Jacksonville, Florida, United States of America; 9 Department of Laboratory Medicine and Pathology, Mayo Clinic, Rochester, Minnesota, United States of America; 10 Department of Molecular Microbiology and Immunology, Johns Hopkins Bloomberg School of Public Health, Baltimore, Maryland, United States of America; 11 Department of Epidemiology and Biostatistics, College of Human Medicine, Michigan State University, East Lansing, Michigan, United States of America; 12 Department of Pediatrics and Human Development, College of Human Medicine, Michigan State University, East Lansing, Michigan, United States of America; 13 Department of Pathology, Duke University, Durham, North Carolina, United States of America; 14 New York Blood Center Enterprises, New York City, New York, United States of America; 15 Vitalant Research Institute, Denver, Colorado, United States of America; 16 University of Colorado School of Medicine, Aurora, Colorado, United States of America; 17 Geisel School of Medicine at Dartmouth, Lebanon, New Hampshire, United States of America; 18 Vitalant, Scottsdale, Arizona, United States of America; 19 American Red Cross, Washington, District of Columbia, United States of America; 20 Center for Biologics Evaluation and Research, U. S. Food and Drug Administration, Silver Spring, Maryland, United States of America; 21 Bloodworks Northwest, Seattle, Washington, United States of America; 22 Department of Cardiovascular Medicine, Mayo Clinic, Rochester, Minnesota, United States of America; 23 Versiti, Indianapolis, Indiana, United States of America; 24 Department of Systems and Computational Biology, Albert Einstein College of Medicine, New York City, New York, United States of America; 25 Division of Pulmonary and Critical Care Medicine, Department of Internal Medicine, Mayo Clinic, Rochester, Minnesota, United States of America; 26 Division of Infectious Diseases, Department of Internal Medicine, Mayo Clinic, Phoenix, Arizona, United States of America; 27 School of Sustainability, Arizona State University, Tempe, Arizona, United States of America; 28 Department of Kinesiology, Indiana University, Bloomington, Indiana, United States of America; Sanquin Blood Bank, NETHERLANDS

## Abstract

**Background:**

The United States (US) Expanded Access Program (EAP) to coronavirus disease 2019 (COVID-19) convalescent plasma was initiated in response to the rapid spread of severe acute respiratory syndrome coronavirus 2 (SARS-CoV-2), the causative agent of COVID-19. While randomized clinical trials were in various stages of development and enrollment, there was an urgent need for widespread access to potential therapeutic agents. The objective of this study is to report on the demographic, geographical, and chronological characteristics of patients in the EAP, and key safety metrics following transfusion of COVID-19 convalescent plasma.

**Methods and findings:**

Mayo Clinic served as the central institutional review board for all participating facilities, and any US physician could participate as a local physician–principal investigator. Eligible patients were hospitalized, were aged 18 years or older, and had—or were at risk of progression to—severe or life-threatening COVID-19; eligible patients were enrolled through the EAP central website. Blood collection facilities rapidly implemented programs to collect convalescent plasma for hospitalized patients with COVID-19. Demographic and clinical characteristics of all enrolled patients in the EAP were summarized. Temporal patterns in access to COVID-19 convalescent plasma were investigated by comparing daily and weekly changes in EAP enrollment in response to changes in infection rate at the state level. Geographical analyses on access to convalescent plasma included assessing EAP enrollment in all national hospital referral regions, as well as assessing enrollment in metropolitan areas and less populated areas that did not have access to COVID-19 clinical trials. From April 3 to August 23, 2020, 105,717 hospitalized patients with severe or life-threatening COVID-19 were enrolled in the EAP. The majority of patients were 60 years of age or older (57.8%), were male (58.4%), and had overweight or obesity (83.8%). There was substantial inclusion of minorities and underserved populations: 46.4% of patients were of a race other than white, and 37.2% of patients were of Hispanic ethnicity. Chronologically and geographically, increases in the number of both enrollments and transfusions in the EAP closely followed confirmed infections across all 50 states. Nearly all national hospital referral regions enrolled and transfused patients in the EAP, including both in metropolitan and in less populated areas. The incidence of serious adverse events was objectively low (<1%), and the overall crude 30-day mortality rate was 25.2% (95% CI, 25.0% to 25.5%). This registry study was limited by the observational and pragmatic study design that did not include a control or comparator group; thus, the data should not be used to infer definitive treatment effects.

**Conclusions:**

These results suggest that the EAP provided widespread access to COVID-19 convalescent plasma in all 50 states, including for underserved racial and ethnic minority populations. The study design of the EAP may serve as a model for future efforts when broad access to a treatment is needed in response to an emerging infectious disease.

**Trial registration:**

ClinicalTrials.gov NCT#: NCT04338360.

## Abstract 2

In an observational study of registry data, Jonathon Senefeld and colleagues study factors related to patient enrollment in the Expanded Access Program for use of COVID-19 convalescent plasma in the United States.

## Author summary

**Why was this study done?:**

- There was a public health need to provide expedited and broad access to convalescent plasma for the treatment of coronavirus disease 2019 (COVID-19) during the early stages of this public health emergency in the United States.
- Convalescent plasma was initially administered through regulatory pathways that required per-patient approval, resulting in substantial administrative time.
- The Expanded Access Program (EAP) was initiated to provide broad access to COVID-19 convalescent plasma and to provide a framework for standardized collection of data describing the safety profile of convalescent plasma.

**What did the researchers do and find?:**

- The EAP provided rapid and broad access to convalescent plasma throughout the US and some US territories and was effective at providing therapy for demographic groups that were severely affected by COVID-19.
- In addition, the data provide evidence supporting that transfusion of convalescent plasma is safe in hospitalized patients with COVID-19.

**What do these findings mean?:**

- The study design of the EAP may serve as an example for future efforts in response to a rapidly developing infectious disease when broad access to a treatment is needed in areas and demographic groups that are often poorly represented in clinical trials.

## Introduction

Severe acute respiratory syndrome coronavirus 2 (SARS-CoV-2), the causative agent of coronavirus disease 2019 (COVID-19), spread rapidly across the United States (US) after confirmation of the first cases of COVID-19 in the US in December 2019 and January 2020 [1]. By March of 2020, community transmission was occurring in major metropolitan areas in the Northeast US, where hospitals became overwhelmed with admissions for severe or life-threatening COVID-19 [1]. Although most of those who are infected have few or no symptoms despite high SARS-CoV-2 viral loads [2], approximately 2% to 7% [3–5] develop hypoxemia and severe COVID-19 leading to hospitalization and the need for supplemental oxygen support [6]. Severe cases of COVID-19 can lead to respiratory failure, which is among the leading causes of death in persons with COVID-19 [7].

The treatment of patients with COVID-19 is primarily supportive [8]. During the early stages of this public health emergency, evidence-based treatments were few, but immunomodulatory agents and antivirals were viewed as promising therapeutic strategies for patients with COVID-19 [9,10]. Passive immunotherapy using convalescent plasma or serum had been used previously to treat diverse infectious diseases [11–13], including severe acute respiratory syndrome coronavirus 1 infection (SARS-CoV-1) [14]. Further, early studies undertaken during the COVID-19 pandemic suggested the potential efficacy of convalescent plasma in the treatment of COVID-19 [15–17].

In late March and early April of 2020, COVID-19 convalescent plasma began to be administered to patients under single-patient emergency investigational new drug (eIND) applications while randomized clinical trials of COVID-19 convalescent plasma were in various stages of development and enrollment. Neither the single eIND process nor the speed at which clinical trials could be implemented was meeting the need to provide access to COVID-19 convalescent plasma for most patients. For example, one institution in New York City had 45 eIND applications submitted to and approved by the US Food and Drug Administration (FDA) in a 2-week period in late March 2020 [18]. The single-patient eIND application process requires substantial administrative support from local institutions and the US FDA, limiting widespread access for patients to convalescent plasma [19,20]. Additionally, clinical trials often have inclusion criteria that are restricted to a specific geographical region or disease status (e.g., hospitalized, but not with severe disease) and have exclusion criteria (e.g., prisoners or recipients of solid organ transplant). Thus, a different regulatory pathway for obtaining access to COVID-19 convalescent plasma and comprehensively studying the safety of convalescent plasma for the treatment of COVID-19 was needed.

To provide access to COVID-19 convalescent plasma treatment and provide a framework for standardized safety data collection, Mayo Clinic initiated the Expanded Access Program (EAP) for COVID-19 convalescent plasma. The primary objective of the EAP was to provide access to convalescent plasma for hospitalized patients in the US with severe or life-threatening COVID-19 [21]. The EAP started as a national registry approved to register 5,000 patients, but due to the extraordinary national demand for COVID-19 convalescent plasma, enrollment goals were extended in collaboration with the US FDA and the Biomedical Advanced Research and Development Authority (BARDA), with the aim of the EAP becoming a broad national program obviating the need for individual-patient investigational new drug (IND) applications. We herein assess the extent to which the EAP was successful in terms of providing access to COVID-19 convalescent plasma by presenting demographic, geographical, and chronological characteristics of patients in the EAP alongside publicly available data of state-level patterns in COVID-19. Additionally, we analyzed key safety metrics following transfusion of convalescent plasma.

## Methods

As described previously [22–24], the EAP was a national registry for hospitalized patients with COVID-19. Collaborative support was provided by the US BARDA and FDA; funding to support the study infrastructure and study-related costs at participating sites was provided under contract from BARDA. Mayo Clinic served as the academic research organization coordinating the national registry. The Mayo Clinic institutional review board (IRB), the central IRB for the registry, approved the protocol (IRB #20–0033412, ClinicalTrials.gov NCT04338360) and all amendments, and provided regulatory oversight for all sites and investigators. The principal investigator (MJJ) was the regulatory sponsor. A data and safety monitoring board oversaw the safety analyses and advised the regulatory sponsor and the Mayo Clinic IRB on risk. Study data were deposited with the US FDA.

The study used a prospective protocol and statistical analysis plan (as previously described [22]), with changes to both plans during the study period associated with the EAP. Full details of the study design, conduct, oversight, and analyses are provided in the protocol and statistical analysis plan (S1 Text). This study is reported as per the Strengthening the Reporting of Observational Studies in Epidemiology (STROBE) guideline (S1 Checklist).

### Patients

Patients were eligible for enrollment in the EAP if they were aged 18 years or older, were hospitalized with a laboratory-confirmed diagnosis of or suspected/probable infection with SARS-CoV-2, and either had or were judged by a healthcare provider to be at high risk of progression to severe or life-threatening COVID-19. Severe COVID-19 was defined by 1 or more of the following: dyspnea, respiratory frequency ≥ 30/minute, blood oxygen saturation ≤ 93%, ratio of partial pressure of arterial oxygen to fraction of inspired oxygen < 300, and lung infiltrates > 50% within 24 to 48 hours of hospital admission. Life-threatening COVID-19 was defined as 1 or more of the following: respiratory failure, septic shock, and multiple organ dysfunction or failure. To maximize access to COVID-19 convalescent plasma, no exclusion criteria were used, thereby enabling access for populations of vulnerable adults who may not be eligible for clinical trials, including pregnant women and prisoners.

### Enrollment

All hospitals and acute care facilities in the US and its territories, and any physician licensed in the US, were allowed to register for participation provided they agreed to adhere to the treatment protocol, which was available online [21], as well as US FDA and state regulations. All patient registration was facilitated through the central study website [25]. A single consent form, available in 8 languages, was used by all participating sites. Prior to patient enrollment, written informed consent was obtained from the patient or a legally authorized representative, or by means of an emergency consent process for patients in a condition that warranted this process. Criteria for emergency consent were consistent with the federal regulations governing emergency consent [26]. Early COVID-19 convalescent plasma safety reports and details on convalescent plasma transfusion antibody titers have been described elsewhere [22–24].

### Distribution and transfusion of COVID-19 convalescent plasma

Eligibility for donation of COVID-19 convalescent plasma was established by the US FDA. In brief, convalescent plasma was donated by individuals with evidence of past SARS-CoV-2 infection, as determined by a positive molecular diagnostic test for COVID-19 (i.e., at time of illness) or a positive serological test. A minimum antibody titer against SARS-CoV-2 was not required for convalescent plasma administration under the EAP, in part because there was no readily deployable assay early in the pandemic. Donation was required to be at least 14 days following resolution of COVID-19 symptoms. The eligibility criteria for donation of convalescent plasma changed over the course of the EAP, including relaxation of a requirement for negative testing (e.g., nasopharyngeal swab) for those donating less than 28 days after resolution of symptoms. Convalescent plasma donors had to satisfy all requirements for allogeneic blood donation, including measures to mitigate transfusion-related acute lung injury (TRALI).

COVID-19 convalescent plasma was collected by US FDA–licensed or–registered blood collectors (i.e., blood centers or hospital-based collection facilities) using the standard blood collection center procedures for plasma collection that predated COVID-19. The units of convalescent plasma were labeled using a facility-specific ISBT 128 code, which enabled tracking of the blood product from source to patient. After a patient was enrolled, convalescent plasma was ordered directly from a participating blood collector and transfused in accordance with the participating institutions’ transfusion guidelines. Initially, the EAP protocol restricted transfusion to a maximum volume of 400 mL of ABO-compatible convalescent plasma. On May 23, 2020, it became permissible to follow institutional transfusion guidelines (e.g., transfusion of group O units with low-titer anti-A), and repeated dosing of convalescent plasma was allowed.

The units of convalescent plasma were tracked from collection through distribution and transfusion. This allowed for comparison of patient enrollment against utilization by location over time. The origin and the distribution of convalescent plasma were mapped using the facility identification code embedded in the ISBT 128 code label of the units of COVID-19 convalescent plasma.

### Study data

Demographic and clinical characteristics of enrolled patients were collected using the Research Electronic Data Capture (REDCap) system (version 9.1.15–10.0.33; Vanderbilt University, Nashville, TN) [27,28]. The online case report forms were designed to optimize convenience. Race (American Indian or Alaska Native, Asian, Black or African American, Native Hawaiian or Other Pacific Islander, white) and ethnicity (Hispanic/Latino or not Hispanic/Latino) were reported in categories by site personnel in a manner consistent with guidelines provided by the US Office of Management and Budget [29].

The database was updated as needed to fulfill the requirements of the EAP IRB and the data collection requirements of BARDA. As the original goal of data collection was to determine safety among 5,000 patients, updates were needed to capture additional clinical data as enrollment expanded and the study progressed. Additionally, in response to surges in COVID-19 infection rates, data collection instruments were simplified by requiring less detailed demographic and clinical information about transfused patients. Enrollment into the EAP was stopped after the US FDA issued an emergency use authorization (EUA) for COVID-19 convalescent plasma on August 23, 2020. Data clarification requests were sent to participating investigators as needed until the database was locked to further data changes on December 16, 2020. All versions of the study protocol, case report forms with completion instructions, and the informed consent form are publicly available on the study website [25].

### COVID-19 epidemiological data sources

In order to contextualize whether the patients enrolled in the EAP were reflective of the US population, race and ethnicity data for each state and US territory were retrieved from the US Census Bureau [30], using the same race and ethnicity categories that were collected in the EAP. Confirmed COVID-19 infection rates per day for each US state were obtained from the New York Times database [31]. Hospital referral regions are regional healthcare markets defined by where most residents within that region have their hospitalization stays. The 306 hospital referral regions in the US were retrieved from the Dartmouth Atlas of Health Care [32]. Data on region type (metropolitan, micropolitan, or neither) were obtained from US Census Bureau data [30] and the 2010 Office of Management and Budget standards that define metropolitan and micropolitan areas based on statistical assessments [33]. Micropolitan areas were defined as areas with a population of at least 10,000 and less than 50,000 residents. The US regions of Northeast, South, Midwest, and West were delineated using commonly used regions [34]. Characteristics of US hospitals were retrieved from American Hospital Directory [35] and the Centers for Medicare & Medicaid Services [36]. The potential limitations of the data sources are described in the Discussion.

#### Serious transfusion reactions

All serious transfusion reactions were reported by the treating physicians and independently adjudicated over the course of the study by the IND sponsor and trained designees using National Healthcare Safety Network Biovigilance Component Hemovigilance Module Surveillance Protocol criteria [37]. Serious transfusion reactions were defined as transfusion-associated circulatory overload (TACO), TRALI, severe allergic reaction, hypotensive reaction, or death. By definition, all serious transfusion reactions occurred within 6 hours of the COVID-19 convalescent plasma transfusion. The attribution categories used for evaluating the relatedness of serious transfusion reactions to COVID-19 convalescent plasma transfusion included unrelated, possibly related, probably related, and definitely related. Serious transfusion reactions were collected using case report forms completed 4 hours and 7 days after transfusion, with additional forms used to report more serious adverse event information when needed.

#### Statistical considerations

To provide a comprehensive report of enrollment data for the EAP program, descriptive statistics are presented for demographic and clinical variables of interest. To examine enrollment in the EAP over time, dot plots are used to show the number of enrollments, for each US state individually and aggregated by region, by day of the study. Additionally, EAP enrollment was compared to the number of confirmed COVID-19 cases per state over the duration of the study. During the window of EAP enrollment, a moving 7-day average was calculated for daily enrollments and COVID-19 cases within each state that enrolled more than 10 patients in total in the EAP. To compare and visualize relative patterns, these averages were scaled between 0 (lowest cases/enrollments) and 1 (peak cases/enrollments) and overlaid on a geofaceted graph. The geofaceted graph contains 1 cell for each US state that is placed at approximately the same location on the graph as the corresponding geographical location of the state on a map. Differences in geographical access to convalescent plasma through the EAP were assessed by examining enrollment across micropolitan and metropolitan areas and the number of hospitals enrolling patients in each hospital referral region in the US. Crude mortality (observed number of deaths divided by the number of transfused patients) is presented across a range of patient and region characteristics. For this analysis, crude mortality was summarized with 95% confidence intervals; no tests for differences among or between levels were performed. All data were processed using R version 3.6.2.

## Results

### Enrolled patients

#### Enrolled patient characteristics

From April 3 to August 23, 2020, 105,717 hospitalized patients with severe or life-threatening COVID-19 were enrolled in the EAP, and approximately 95,000 patients were transfused with COVID-19 convalescent plasma. The EAP halted enrollment forthwith after the US FDA issued an EUA for COVID-19 convalescent plasma on August 23, 2020, stating that the totality of scientific evidence indicated that convalescent plasma was safe [23,24] and a potentially promising therapeutic treatment [38]. This authorization enabled physicians to use COVID-19 convalescent plasma without requesting eIND or IND permission and obviated the need for access to convalescent plasma via the EAP.

Enrolled patients’ demographic characteristics (including age, sex, race, ethnicity) and clinical characteristics at the time of transfusion are shown in Table 1. The majority of patients were 60 years of age or older (57.8%), were male (58.4%), had overweight or obesity (83.8%), and had never smoked (69.7%). There was inclusion of minorities and underserved populations; 46.4% of patients were of a race other than white, and 37.2% of patients were of Hispanic ethnicity. Preexisting conditions present among enrolled patients, and concomitant medications, are also displayed in Table 1. Of those patients enrolled, 61.8% had severe or life-threatening COVID-19, 42.3% were in the intensive care unit (ICU), and 19.8% had received intubation or a higher level of respiratory support at the time of transfusion. A small proportion of patients (3.9%) had no form of hospital respiratory support prior to infusion. A large percentage of patients had dyspnea (75.7%), oxygen saturation ≤ 93% (75.0%), and acute respiratory failure (60.6%). Many patients were prescribed steroids (65.7%), azithromycin (49.0%), and remdesivir (37.6%) during their hospital stay. The median number of days between diagnosis of COVID-19 and the first transfusion was 4 days (interquartile range, 2–8 days), and nearly half of transfused patients (45.0%) received convalescent plasma within 3 days of COVID-19 diagnosis, often during hospital admission.

**Table 1:** Characteristics and crude mortality rates of patients with COVID-19 who were enrolled in the US Expanded Access Program for convalescent plasma.

Characteristic	Transfused patients	Not transfused/unreported	All enrolled patients	30-day crude mortality (95% CI)^a^
**Consent type**				
Patient signed	65,067/94,254 (69.0)	7,482/11,406 (65.6)	72,549/105,660 (68.7)	17.93% (17.64%, 18.23%)
LAR/surrogate signed	24,634/94,254 (26.1)	3,293/11,406 (28.9)	27,927/105,660 (26.4)	42.00% (41.38%, 42.62%)
Emergency exception	4,553/94,254 (4.8)	631/11,406 (5.5)	5,184/105,660 (4.9)	38.59% (37.18%, 40.01%)
**Age at enrollment (years)**				
18 to 19	127/94,287 (0.1)	22/11,430 (0.2)	149/105,717 (0.1)	8.66% (4.91%, 14.85%)
20 to 29	2,367/94,287 (2.5)	320/11,430 (2.8)	2,687/105,717 (2.5)	6.20% (5.30%, 7.25%)
30 to 39	5,978/94,287 (6.3)	816/11,430 (7.1)	6,794/105,717 (6.4)	7.57% (6.92%, 8.27%)
40 to 49	11,703/94,287 (12.4)	1,476/11,430 (12.9)	13,179/105,717 (12.5)	11.56% (10.99%, 12.15%)
50 to 59	19,488/94,287 (20.7)	2,301/11,430 (20.1)	21,789/105,717 (20.6)	16.66% (16.14%, 17.19%)
60 to 69	23,632/94,287 (25.1)	2,673/11,430 (23.4)	26,305/105,717 (24.9)	27.03% (26.47%, 27.60%)
70 to 79	19,351/94,287 (20.5)	2,234/11,430 (19.5)	21,585/105,717 (20.4)	36.25% (35.57%, 36.93%)
80 to 89	9,617/94,287 (10.2)	1,263/11,430 (11.0)	10,880/105,717 (10.3)	43.81% (42.82%, 44.80%)
90 to 99	1,970/94,287 (2.1)	308/11,430 (2.7)	2,278/105,717 (2.2)	47.48% (45.27%, 49.69%)
100+	54/94,287 (0.1)	17/11,430 (0.1)	71/105,717 (0.1)	40.74% (28.68%, 54.03%)
**Sex**				
Female		4,732/11,430 (41.4)	43,544/105,717 (41.2)	23.30% (22.88%, 23.72%)
Male	55,109/94,287 (58.4)	6,652/11,430 (58.2)	61,761/105,717 (58.4)	26.61% (26.24%, 26.98%)
Intersex	141/94,287 (0.1)	16/11,430 (0.1)	157/105,717 (0.1)	22.70% (16.56%, 30.28%)
Transgender^b^	129/94,287 (0.1)	15/11,430 (0.1)	144/105,717 (0.1)	20.93% (14.80%, 28.74%)
Prefer not to disclose	96/94,287 (0.1)	15/11,430 (0.1)	111/105,717 (0.1)	19.79% (13.05%, 28.86%)
**Weight status** ^**c**^				
Underweight	1,111/91,920 (1.2)	194/10,925 (1.8)	1,305/102,845 (1.3)	33.24% (30.53%, 36.07%)
Normal weight	13,551/91,920 (14.7)	1,746/10,925 (16.0)	15,297/102,845 (14.9)	31.43% (30.65%, 32.22%)
Overweight	25,460/91,920 (27.7)	3,062/10,925 (28.0)	28,522/102,845 (27.7)	26.99% (26.45%, 27.54%)
Class 1 obesity	22,782/91,920 (24.8)	2,568/10,925 (23.5)	25,350/102,845 (24.6)	24.00% (23.45%, 24.56%)
Class 2 obesity	13,734/91,920 (14.9)	1,547/10,925 (14.2)	15,281/102,845 (14.9)	21.87% (21.19%, 22.57%)
Class 3 obesity	15,282/91,920 (16.6)	1,808/10,925 (16.5)	17,090/102,845 (16.6)	20.17% (19.54%, 20.82%)
**Race**				
American Indian or Alaska Native alone	1,346/94,286 (1.4)	88/11,429 (0.8)	1,434/105,715 (1.4)	27.06% (24.76%, 29.50%)
Asian alone	3,018/94,286 (3.2)	414/11,429 (3.6)	3,432/105,715 (3.2)	25.53% (24.01%, 27.12%)
Black or African American alone	16,988/94,286 (18.0)	2,237/11,429 (19.6)	19,225/105,715 (18.2)	24.71% (24.06%, 25.36%)
Native Hawaiian or Other Pacific Islander alone	549/94,286 (0.6)	56/11,429 (0.5)	605/105,715 (0.6)	16.76% (13.87%, 20.11%)
Two or more races	427/94,286 (0.5)	56/11,429 (0.5)	483/105,715 (0.5)	24.59% (20.74%, 28.89%)
White alone	50,972/94,286 (54.1)	5,715/11,429 (50.0)	56,687/105,715 (53.6)	26.09% (25.71%, 26.48%)
Other or unknown	20,986/94,286 (22.3)	2,863/11,429 (25.1)	23,849/105,715 (22.6)	23.61% (23.04%, 24.19%)
**Ethnicity**				
Hispanic/Latino	34,807/94,287 (36.9)	4,528/11,430 (39.6)	39,335/105,717 (37.2)	24.27% (23.82%, 24.72%)
Not Hispanic/Latino	59,480/94,287 (63.1)	6,902/11,430 (60.4)	66,382/105,717 (62.8)	25.79% (25.44%, 26.14%)
**Blood type**				
O−	3,795/94,287 (4.0)	428/11,430 (3.7)	4,223/105,717 (4.0)	28.03% (26.62%, 29.48%)
O+	43,655/94,287 (46.3)	4,934/11,430 (43.2)	48,589/105,717 (46.0)	25.21% (24.81%, 25.62%)
A−	3,301/94,287 (3.5)	372/11,430 (3.3)	3,673/105,717 (3.5)	27.33% (25.84%, 28.88%)
A+	28,665/94,287 (30.4)	3,217/11,430 (28.1)	31,882/105,717 (30.2)	24.82% (24.32%, 25.32%)
B−	892/94,287 (0.9)	137/11,430 (1.2)	1,029/105,717 (1.0)	25.96% (23.18%, 28.93%)
B+	10,868/94,287 (11.5)	1,693/11,430 (14.8)	12,561/105,717 (11.9)	24.62% (23.82%, 25.44%)
AB−	310/94,287 (0.3)	74/11,430 (0.6)	384/105,717 (0.4)	30.74% (25.86%, 36.10%)
AB+	2,801/94,287 (3.0)	575/11,430 (5.0)	3,376/105,717 (3.2)	24.86% (23.29%, 26.50%)
**Enrollment month**				
April	7,130/94,287 (7.6)	1,589/11,430 (13.9)	8,719/105,717 (8.2)	36.04% (34.93%, 37.16%)
May	14,425/94,287 (15.3)	1,448/11,430 (12.7)	15,873/105,717 (15.0)	28.73% (28.00%, 29.48%)
June	16,603/94,287 (17.6)	1,362/11,430 (11.9)	17,965/105,717 (17.0)	22.30% (21.67%, 22.94%)
July	34,506/94,287 (36.6)	4,815/11,430 (42.1)	39,321/105,717 (37.2)	24.83% (24.38%, 25.29%)
August	21,623/94,287 (22.9)	2,216/11,430 (19.4)	23,839/105,717 (22.5)	22.19% (21.64%, 22.75%)
**COVID19 severity at enrollment**				
Currently has severe/life-threatening COVID-19	58,478/94,287 (62.0)	6,858/11,430 (60.0)	65,336/105,717 (61.8)	30.00% (29.63%, 30.38%)
At high risk of progression to severe/life-threatening disease (judged by provider)	35,809/94,287 (38.0)	4,572/11,430 (40.0)	40,381/105,717 (38.2)	17.41% (17.02%, 17.81%)
**Smoking status**				
Current smoker	1,565/30,532 (5.1)	125/2,299 (5.4)	1,690/32,831 (5.1)	20.77% (18.83%, 22.85%)
Past smoker	7,727/30,532 (25.3)	523/2,299 (22.7)	8,250/32,831 (25.1)	32.42% (31.38%, 33.47%)
Never smoked	21,240/30,532 (69.6)	1,651/2,299 (71.8)	22,891/32,831 (69.7)	21.49% (20.94%, 22.05%)
**Highest level of hospital respiratory support prior to transfusion**				
None	3,613/93,430 (3.9)		3,613/93,430 (3.9)	7.48% (6.67%, 8.39%)
Oxygen supplementation	34,965/93,430 (37.4)		34,965/93,430 (37.4)	11.32% (10.99%, 11.66%)
Noninvasive positive-pressure ventilation (NIPPV)	36,350/93,430 (38.9)		36,350/93,430 (38.9)	28.37% (27.91%, 28.84%)
Mechanical ventilation/intubation	18,209/93,430 (19.5)		18,209/93,430 (19.5)	48.88% (48.15%, 49.60%)
Extracorporeal membrane oxygenation (ECMO)	293/93,430 (0.3)		293/93,430 (0.3)	31.40% (26.35%, 36.93%)
**Intensive care unit care prior to infusion**				
No	54,255/94,036 (57.7)		54,255/94,036 (57.7)	16.16% (15.85%, 16.47%)
Yes	39,781/94,036 (42.3)		39,781/94,036 (42.3)	37.60% (37.12%, 38.07%)
**Severe COVID-19 symptoms**				
Dyspnea				
No	14,007/58,478 (24.0)	1,843/6,858 (26.9)	15,850/65,336 (24.3)	37.42% (36.62%, 38.22%)
Yes	44,471/58,478 (76.0)	5,015/6,858 (73.1)	49,486/65,336 (75.7)	27.67% (27.25%, 28.09%)
Respiratory frequency ≥ 30/minute				
No	35,838/58,478 (61.3)	3,978/6,858 (58.0)	39,816/65,336 (60.9)	28.00% (27.54%, 28.47%)
Yes	22,640/58,478 (38.7)	2,880/6,858 (42.0)	25,520/65,336 (39.1)	33.18% (32.57%, 33.80%)
Blood oxygen saturation ≤ 93%				
No	14,489/58,478 (24.8)	1,868/6,858 (27.2)	16,357/65,336 (25.0)	33.73% (32.96%, 34.50%)
Yes	43,989/58,478 (75.2)	4,990/6,858 (72.8)	48,979/65,336 (75.0)	28.78% (28.36%, 29.20%)
PaO2:FiO2 ratio < 300				
No	44,020/58,478 (75.3)	4,775/6,858 (69.6)	48,795/65,336 (74.7)	27.53% (27.12%, 27.95%)
Yes	14,458/58,478 (24.7)	2,083/6,858 (30.4)	16,541/65,336 (25.3)	37.53% (36.74%, 38.32%)
Lung infiltrates > 50% within 24 to 48 hours				
No	36,930/58,478 (63.2)	4,017/6,858 (58.6)	40,947/65,336 (62.7)	28.05% (27.60%, 28.52%)
Yes	21,548/58,478 (36.8)	2,841/6,858 (41.4)	24,389/65,336 (37.3)	33.35% (32.72%, 33.99%)
**Life-threatening COVID-19 symptoms**				
Respiratory failure				
No	23,396/58,478 (40.0)	2,323/6,858 (33.9)	25,719/65,336 (39.4)	20.49% (19.97%, 21.01%)
Yes	35,082/58,478 (60.0)	4,535/6,858 (66.1)	39,617/65,336 (60.6)	36.36% (35.85%, 36.86%)
Septic shock				
No	54,554/58,478 (93.3)	6,160/6,858 (89.8)	60,714/65,336 (92.9)	28.58% (28.20%, 28.96%)
Yes	3,924/58,478 (6.7)	698/6,858 (10.2)	4,622/65,336 (7.1)	49.86% (48.29%, 51.43%)
Multiple organ dysfunction or failure				
No	53,537/58,478 (91.6)	5,968/6,858 (87.0)	59,505/65,336 (91.1)	28.27% (27.89%, 28.65%)
Yes	4,941/58,478 (8.4)	890/6,858 (13.0)	5,831/65,336 (8.9)	48.84% (47.45%, 50.24%)
**Preexisting conditions**				
History of lung disease (e.g., COPD, lung cancer)				
No	34,353/41,141 (83.5)	2,503/2,940 (85.1)	36,856/44,081 (83.6)	25.68% (25.22%, 26.15%)
Yes	6,788/41,141 (16.5)	437/2,940 (14.9)	7,225/44,081 (16.4)	32.08% (30.98%, 33.20%)
Cancer other lung cancer				
No	39,242/41,141 (95.4)	2,771/2,940 (94.3)	42,013/44,081 (95.3)	26.35% (25.92%, 26.79%)
Yes	1,899/41,141 (4.6)	169/2,940 (5.7)	2,068/44,081 (4.7)	34.65% (32.54%, 36.82%)
History of cardiovascular conditions				
No	20,771/41,141 (50.5)	1,637/2,940 (55.7)	22,408/44,081 (50.8)	21.51% (20.96%, 22.08%)
Yes	20,370/41,141 (49.5)	1,303/2,940 (44.3)	21,673/44,081 (49.2)	32.06% (31.43%, 32.71%)
HIV-positive				
No	40,803/41,141 (99.2)	2,927/2,940 (99.6)	43,730/44,081 (99.2)	26.73% (26.31%, 27.17%)
Yes	338/41,141 (0.8)	13/2,940 (0.4)	351/44,081 (0.8)	26.92% (22.47%, 31.89%)
HCV-positive				
No	40,781/41,141 (99.1)	2,925/2,940 (99.5)	43,706/44,081 (99.1)	26.68% (26.25%, 27.11%)
Yes	360/41,141 (0.9)	15/2,940 (0.5)	375/44,081 (0.9)	33.61% (28.93%, 38.64%)
On immunosuppressive therapy				
No	39,592/41,141 (96.2)	2,858/2,940 (97.2)	42,450/44,081 (96.3)	26.42% (25.98%, 26.85%)
Yes	1,549/41,141 (3.8)	82/2,940 (2.8)	1,631/44,081 (3.7)	34.93% (32.59%, 37.33%)
Diabetes				
No	24,641/41,141 (59.9)	1,828/2,940 (62.2)	26,469/44,081 (60.0)	24.28% (23.75%, 24.82%)
Yes	16,500/41,141 (40.1)	1,112/2,940 (37.8)	17,612/44,081 (40.0)	30.40% (29.70%, 31.11%)
**Medications during hospital stay**				
ARB				
No	38,129/40,880 (93.3)	2,734/2,925 (93.5)	40,863/43,805 (93.3)	26.99% (26.55%, 27.44%)
Yes	2,751/40,880 (6.7)	191/2,925 (6.5)	2,942/43,805 (6.7)	23.88% (22.33%, 25.51%)
ACE inhibitor				
No	37,351/40,880 (91.4)	2,702/2,925 (92.4)	40,053/43,805 (91.4)	26.97% (26.52%, 27.42%)
Yes	3,529/40,880 (8.6)	223/2,925 (7.6)	3,752/43,805 (8.6)	24.77% (23.38%, 26.22%)
Azithromycin				
No	20,836/40,880 (51.0)	1,499/2,925 (51.2)	22,335/43,805 (51.0)	26.85% (26.25%, 27.45%)
Yes	20,044/40,880 (49.0)	1,426/2,925 (48.8)	21,470/43,805 (49.0)	26.71% (26.10%, 27.33%)
Remdesivir				
No	25,269/40,880 (61.8)	2,076/2,925 (71.0)	27,345/43,805 (62.4)	28.61% (28.06%, 29.17%)
Yes	15,611/40,880 (38.2)	849/2,925 (29.0)	16,460/43,805 (37.6)	23.81% (23.15%, 24.49%)
Steroids				
No	14,036/40,880 (34.3)	1,005/2,925 (34.4)	15,041/43,805 (34.3)	23.45% (22.75%, 24.15%)
Yes	26,844/40,880 (65.7)	1,920/2,925 (65.6)	28,764/43,805 (65.7)	28.52% (27.99%, 29.07%)
Hydroxychloroquine and/or chloroquine				
No	33,557/40,880 (82.1)	2,380/2,925 (81.4)	35,937/43,805 (82.0)	25.14% (24.68%, 25.60%)
Yes	7,323/40,880 (17.9)	545/2,925 (18.6)	7,868/43,805 (18.0)	34.31% (33.23%, 35.40%)
**Census region or territory**				
Midwest	13,454/94,287 (14.3)	1,423/11,430 (12.4)	14,877/105,717 (14.1)	22.27% (21.58%, 22.98%)
Northeast	10,372/94,287 (11.0)	1,173/11,430 (10.3)	11,545/105,717 (10.9)	33.25% (32.35%, 34.16%)
South	52,178/94,287 (55.3)	6,151/11,430 (53.8)	58,329/105,717 (55.2)	24.68% (24.31%, 25.05%)
West	18,103/94,287 (19.2)	2,636/11,430 (23.1)	20,739/105,717 (19.6)	24.28% (23.66%, 24.91%)
US territory	180/94,287 (0.2)	47/11,430 (0.4)	227/105,717 (0.2)	37.99% (31.20%, 45.28%)
**Micro/metropolitan**				
Metropolitan	89,837/94,287 (95.3)	10,790/11,430 (94.4)	100,627/105,717 (95.2)	25.37% (25.09%, 25.66%)
Micropolitan	3,888/94,287 (4.1)	565/11,430 (4.9)	4,453/105,717 (4.2)	22.52% (21.24%, 23.86%)
Neither	562/94,287 (0.6)	75/11,430 (0.7)	637/105,717 (0.6)	20.68% (17.52%, 24.25%)
**Rural referral center**				
No	78,232/93,166 (84.0)	9,699/11,328 (85.6)	87,931/104,494 (84.1)	25.09% (24.79%, 25.39%)
Yes	14,934/93,166 (16.0)	1,629/11,328 (14.4)	16,563/104,494 (15.9)	26.18% (25.48%, 26.89%)
**Sole community hospital**				
No	89,246/93,166 (95.8)	10,731/11,328 (94.7)	99,977/104,494 (95.7)	25.28% (25.00%, 25.57%)
Yes	3,920/93,166 (4.2)	597/11,328 (5.3)	4,517/104,494 (4.3)	24.87% (23.54%, 26.25%)
**Part of a health system**				
No	13,177/93,166 (14.1)	2,107/11,328 (18.6)	15,284/104,494 (14.6)	23.95% (23.22%, 24.68%)
Yes	79,989/93,166 (85.9)	9,221/11,328 (81.4)	89,210/104,494 (85.4)	25.48% (25.18%, 25.79%)
**Major teaching hospital (member of COTH)**				
No	66,531/90,463 (73.5)	8,932/11,008 (81.1)	75,463/101,471 (74.4)	25.07% (24.74%, 25.40%)
Yes	23,932/90,463 (26.5)	2,076/11,008 (18.9)	26,008/101,471 (25.6)	25.66% (25.11%, 26.21%)
**University affiliated**				
No	42,074/93,166 (45.2)	5,763/11,328 (50.9)	47,837/104,494 (45.8)	24.28% (23.87%, 24.70%)
Yes	51,092/93,166 (54.8)	5,565/11,328 (49.1)	56,657/104,494 (54.2)	26.07% (25.69%, 26.45%)

All values are presented as number/total number (percent).

^a^Crude mortality is shown for transfused patients only; 95% confidence intervals (CIs) were estimated using binomial proportions via the Wilson method.

^b^Transgender is a gender-specific term (as opposed to a sex-specific term).

^c^Weight status based on BMI. Underweight: below 18.5 kg/m^2^; normal weight: 18.5–24 kg/m^2^; overweight: 25–29 kg/m^2^; class 1 obesity: 30–34 kg/m^2^; class 2 obesity: 35–39 kg/m^2^; class 3 obesity: 40+ kg/m^2^.

ACE, angiotensin-converting enzyme; ARB, angiotensin II receptor blocker; COPD, chronic obstructive pulmonary disease; COTH, Council of Teaching Hospitals; HCV, hepatitis C virus; LAR, legally authorized representative; PaO2:FiO2, ratio of partial pressure of arterial oxygen to fraction of inspired oxygen.

#### Geospatial patterns in enrollment

Patients were enrolled from each state in the US, the District of Columbia, and the US territories of Puerto Rico and the US Virgin Islands (Table 2). A large percentage of patients were enrolled in the Southern region of the US (55.2%), and most patients enrolled in a hospital within a metropolitan area (95.2%) that was part of a health system (85.4%) and/or was university affiliated (54.2%) (Table 1).

**Table 2:** Tabular summaries of patient enrollment in the US Expanded Access Program (EAP) for convalescent plasma and US COVID-19 cases during the EAP enrollment period, stratified by US state or territory, ordered by enrollments per 1,000 COVID-19-positive cases.

State or territory	EAP summaries			Population and COVID-19-positive ratios		
	Enrolling sites	Enrolled patients (*N =* 105,717)	Transfused patients (*N* = 94,287)	Population COVID-19-positive cases per enrollment	Enrollments per 10,000 people	Enrollments per 1,000 COVID-19-positive cases
Hawaii	7	320	316	25.6	2.3	39.1
South Carolina	37	3,971	3,529	29.6	7.7	33.7
South Dakota	6	427	409	31.3	4.8	31.9
Texas	220	19,378	17,518	32.8	6.7	30.5
District of Columbia	9	349	278	38.4	4.9	26.0
Oklahoma	26	1,416	1,325	41.0	3.6	24.4
Delaware	4	373	367	45.7	3.8	21.9
Georgia	73	5,423	4,919	46.0	5.1	21.7
Connecticut	23	1,022	929	48.3	2.9	20.7
Florida	158	12,575	11,222	49.0	5.9	20.4
Iowa	35	1,298	1,221	49.8	4.1	20.1
Arizona	41	3,960	3,678	50.6	5.4	19.8
North Dakota	7	226	216	51.7	3.0	19.4
Alabama	35	2,409	2,016	51.9	4.9	19.3
Nevada	19	1,302	1,127	52.3	4.2	19.1
Indiana	64	1,745	1,609	53.7	2.6	18.6
Montana	8	133	127	54.3	1.2	18.4
Ohio	77	2,184	2,007	55.2	1.9	18.1
New Hampshire	8	123	90	55.8	0.9	17.9
Kentucky	40	882	834	56.9	2.0	17.6
Tennessee	50	2,590	2,443	57.7	3.8	17.3
New Mexico	12	430	412	58.1	2.1	17.2
Maryland	42	1,815	1,594	58.8	3.0	17.0
California	235	11,874	10,079	59.2	3.0	16.9
Mississippi	19	1,380	1,313	59.3	4.6	16.9
Kansas	22	703	610	61.0	2.4	16.4
New Jersey	66	2,767	2,451	62.0	3.1	16.1
Missouri	41	1,349	1,264	62.8	2.2	15.9
Wisconsin	60	1,191	1,098	66.5	2.0	15.0
Nebraska	16	505	468	67.4	2.6	14.8
Virginia	51	1,748	1,481	68.1	2.0	14.7
Maine	9	61	57	68.6	0.5	14.6
Pennsylvania	90	1,928	1,700	69.0	1.5	14.5
Minnesota	34	1,049	1,007	71.7	1.9	13.9
Illinois	92	3,135	2,709	73.6	2.5	13.6
Washington	34	939	878	76.8	1.2	13.0
New York	109	4,489	4,143	79.1	2.3	12.6
Oregon	16	319	289	81.5	0.8	12.3
Colorado	26	645	627	84.4	1.1	11.8
Utah	8	593	574	86.3	1.8	11.6
Rhode Island	8	247	241	86.6	2.3	11.6
Louisiana	44	1,548	1,351	92.0	3.3	10.9
North Carolina	56	1,771	1,629	94.0	1.7	10.6
Michigan	61	1,065	869	97.5	1.1	10.3
West Virginia	11	102	88	98.6	0.6	10.1
Alaska	3	59	58	100.5	0.8	9.9
Arkansas	16	599	500	101.2	2.0	9.9
Virgin Islands	2	9	6	123.2	0.8	8.1
Massachusetts	36	907	790	133.2	1.3	7.5
Puerto Rico	30	218	181	151.0	0.7	6.6
Idaho	10	156	131	202.5	0.9	4.9
Wyoming	4	9	9	411.6	0.2	2.4
Vermont	1	1	1	1,303.0	0.0	0.8

**Fig 1:**
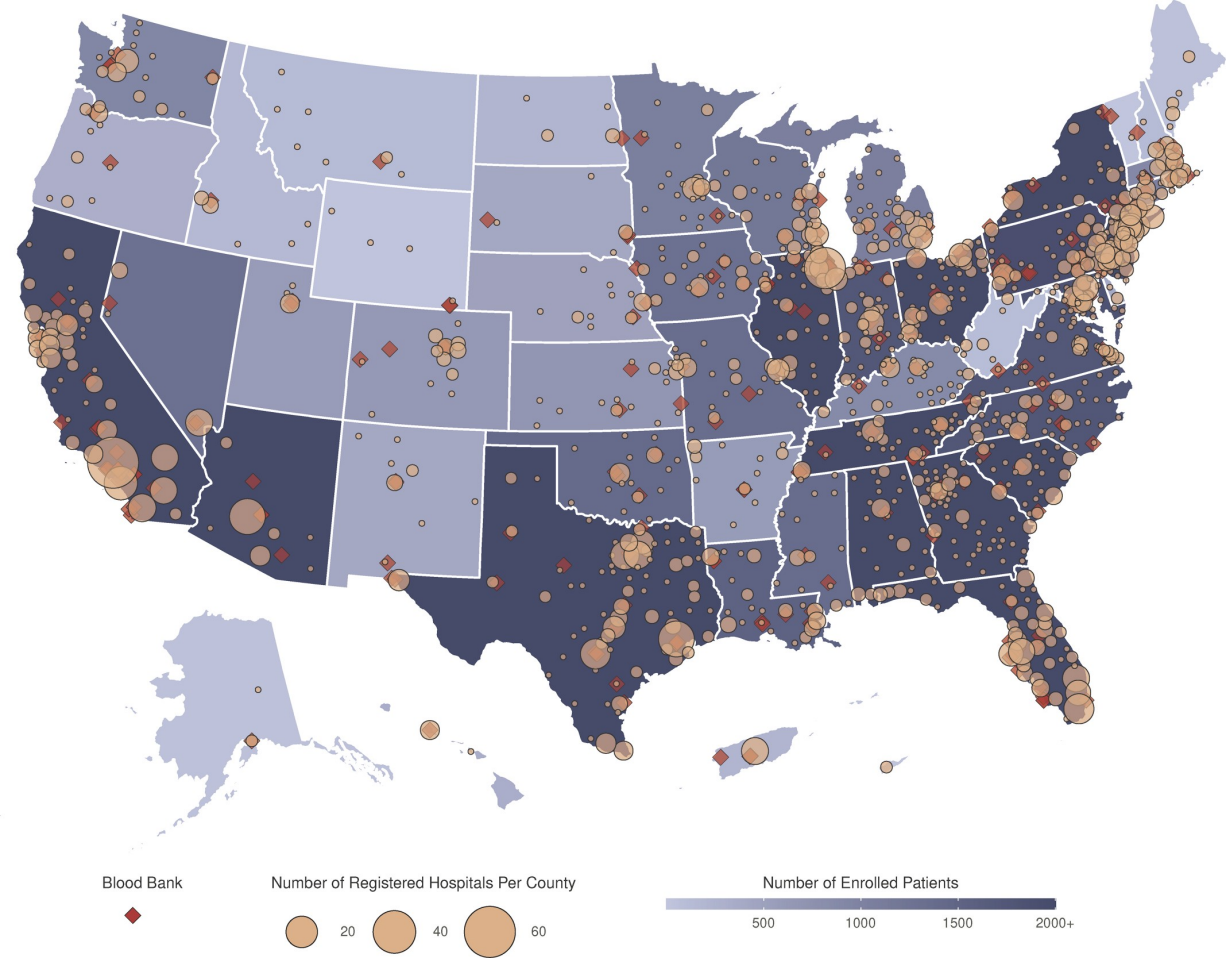
Participation in the US Expanded Access Program (EAP) for convalescent plasma. Choropleth map displaying the number of cumulatively enrolled patients in the EAP within each state of the US and participating territories, with lower enrollment values displayed in a lighter shade of blue and higher enrollment values displayed in a darker shade of blue. Registered acute care facilities are represented as filled yellow circles, with circle size corresponding to the number of registered facilities within the county. Blood collection centers are represented as filled red diamonds. All sites with registered patients were included. The choropleth map does not display Guam or the Northern Mariana Islands. The base layer of the geographical map was created using geographical data retrieved from the US Census Bureau (https://www2.census.gov/geo/tiger/TIGER2019/STATE/). No copyrighted material was used.

**Fig 2:**
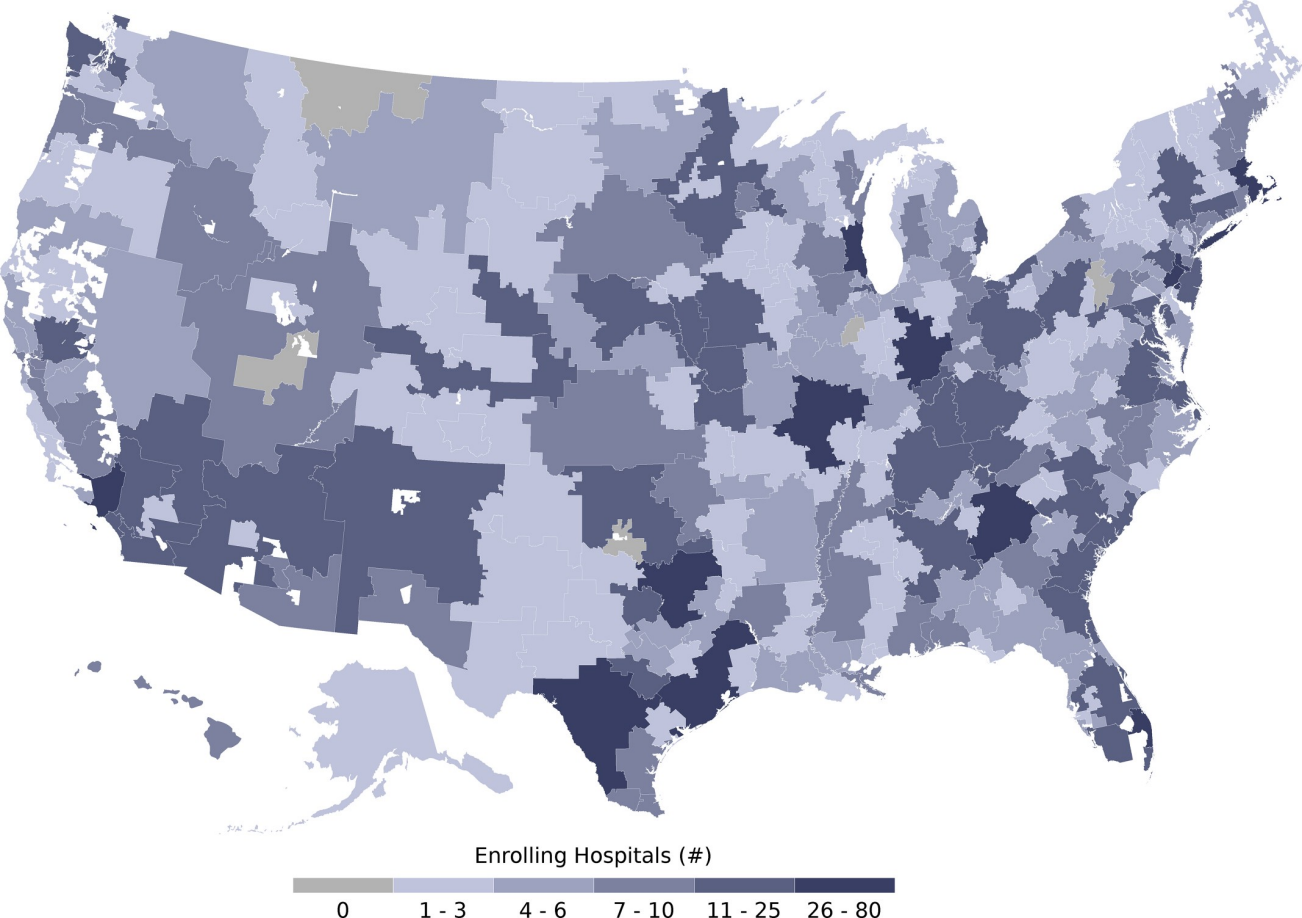
Participation of acute care facilities in the US Expanded Access Program (EAP) for convalescent plasma stratified by US hospital referral region. Choropleth map displaying the number of participating acute care facilities that enrolled patients in the EAP within each hospital referral region—a geographical region that represents a catchment region of patients who get healthcare at similar facilities. Lower numbers of participating acute care facilities are displayed in a lighter hue of blue, and higher numbers of participating acute care facilities are displayed in a darker hue of blue. Hospital referral regions with 0 participating acute care facilities are displayed in grey. Hospital referral regions are not defined in US territories; thus, the choropleth map does not display data from Puerto Rico, the US Virgin Islands, Guam, or the Northern Mariana Islands. The base layer of the geographical map was retrieved from the Dartmouth Atlas Project (https://data.dartmouthatlas.org/supplemental/#boundaries) [32].

Patients were enrolled at 2,211 hospitals and acute care facilities across the US (Fig 1; Table 3). The median number of patients per site was 22 (range, 1 to 1,175). While 2,722 sites were registered, 511 (18.8%) enrolled no patients, 713 (26.2%) enrolled between 1 and 10 patients, and 1,498 (55.0%) enrolled more than 10 patients. Registered sites encompassed nearly all hospital referral regions in the US (Fig 2). Site participation occurred both in metropolitan and non-metropolitan areas and involved different hospital types ranging from community hospitals to major teaching hospitals (Table 3).

**Table 3:** Characteristics of sites that enrolled patients in the US Expanded Access Program (EAP) for convalescent plasma.

Characteristic	Number of sites/total number (percent)
**Census region or territory**	
Midwest	515/2,211 (23.3%)
Northeast	350/2,211 (15.8%)
South	891/2,211 (40.3%)
West	423/2,211 (19.1%)
US territory	32/2,211 (1.4%)
**Micro/metropolitan**	
Metropolitan	1,903/2,211 (86.1%)
Micropolitan	243/2,211 (11.0%)
Neither	65/2,211 (2.9%)
**Urban/rural classification**	
Rural	595/2,081 (28.6%)
Urban	1,486/2,081 (71.4%)
**Sole community hospital**	
No	1,975/2,174 (90.8%)
Yes	199/2,174 (9.2%)
**Rural referral center**	
No	1,824/2,174 (83.9%)
Yes	350/2,174 (16.1%)
**Major teaching hospital (member of COTH)**	
No	1,598/2,042 (78.3%)
Yes	444/2,042 (21.7%)
**University affiliated**	
No	1,120/2,174 (51.5%)
Yes	1,054/2,174 (48.5%)

COTH, Council of Teaching Hospitals.

**Fig 3:**
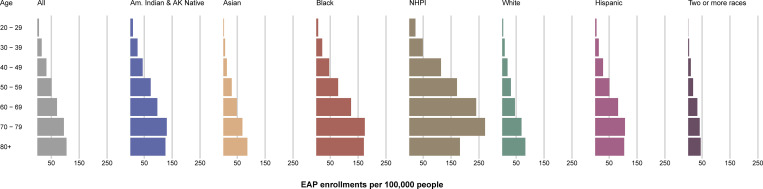
Patient enrollment in the US Expanded Access Program (EAP) stratified by age, race, and ethnicity group, per 100,000 people (from the US census). The length of each colored bar is proportional to the number of patients enrolled in the US EAP within the identified age group (years) and race or ethnicity category. The patient enrollment values are presented relative to analogous categorical data retrieved from the US Census Bureau. Am. Indian & AK Native, American Indian or Alaska Native; NHPI, Native Hawaiian or Other Pacific Islander.

Recognizing the disproportionate effects of the COVID-19 pandemic on minority communities [20], the EAP sought widespread access to convalescent plasma for patients. Patient enrollment stratified by race and ethnicity groups is displayed in Fig 3 and summarized in S1 Table per 100,000 people (from the US census). EAP enrollments were consistent across racial and ethnic groups as well as across age groups (Fig 3).

#### Temporal patterns in enrollment

**Fig 4:**
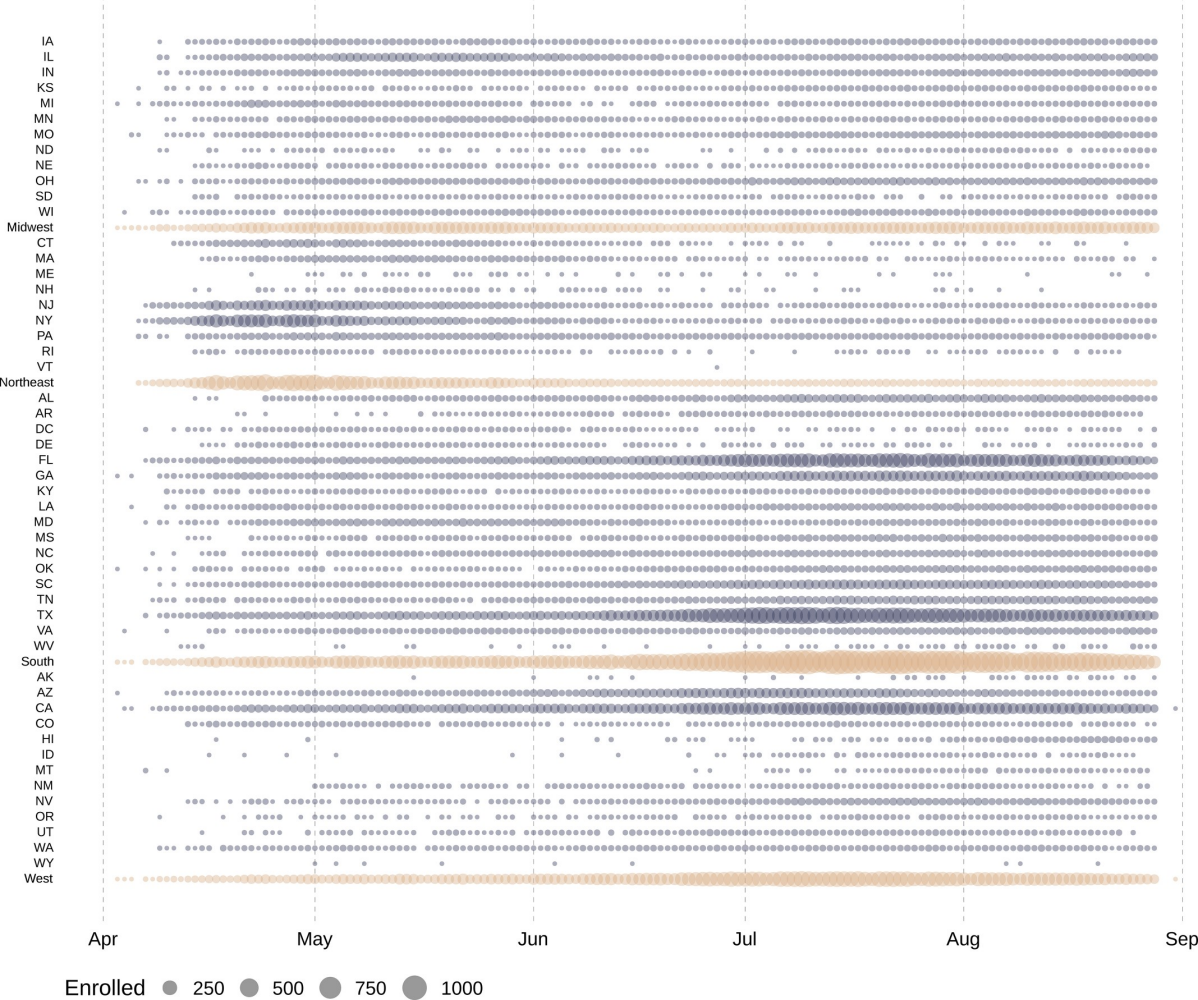
Daily patient enrollment in the US Expanded Access Program. Each circle represents 1 day in which at least 1 patient was enrolled within the indicated US state or region. Grey circles represent daily US state enrollments, and tan circles represent daily US region enrollments. The size of the circle corresponds to the number of daily enrollments within the specified US state or region. States are ordered alphabetically within each US region, followed by the aggregate for each region.

**Fig 5:**
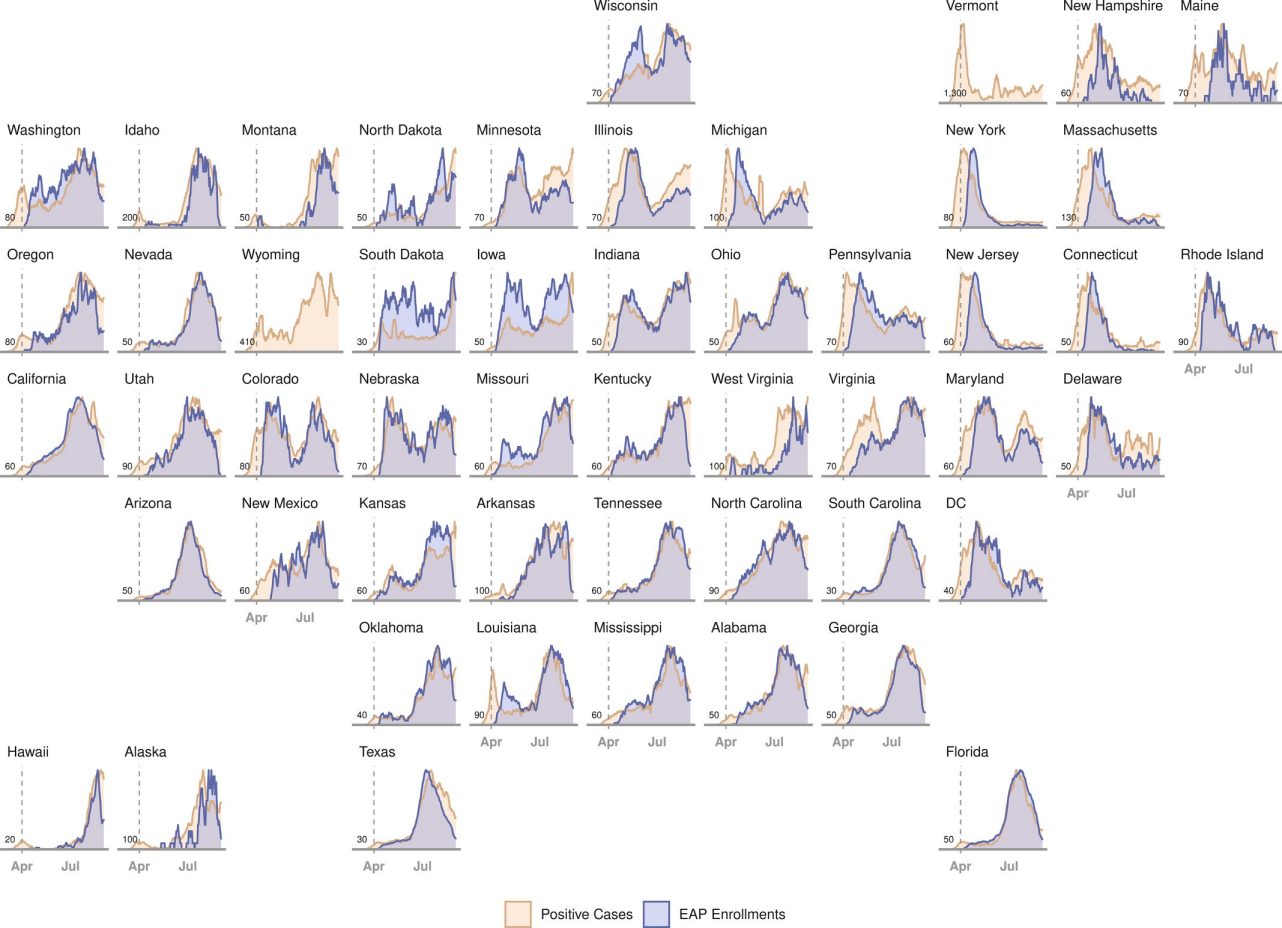
Daily rates of confirmed COVID-19 infections and patient enrollment in the US Expanded Access Program (EAP). Chronological line charts represent the daily number of statewide confirmed COVID-19 infections and EAP patient enrollments sequentially arranged in a geofaceted depiction of the US. Daily rates are presented as a moving average across 7 days, scaled between 0 (least cases/enrollments) and 1 (most cases/enrollments) for any day in each state. Vertical dashed grey lines represent the start date of the EAP (April 3, 2020). Values in the lower left corner of each panel indicate the scaling factor between the 2 plots (cases/enrollments), which approximates the number of COVID-19 cases that contributed to 1 enrollment in the EAP. EAP enrollment data are not presented for Vermont or Wyoming because total enrollments were not greater than 10 patients. DC, District of Columbia.

**Fig 6:**
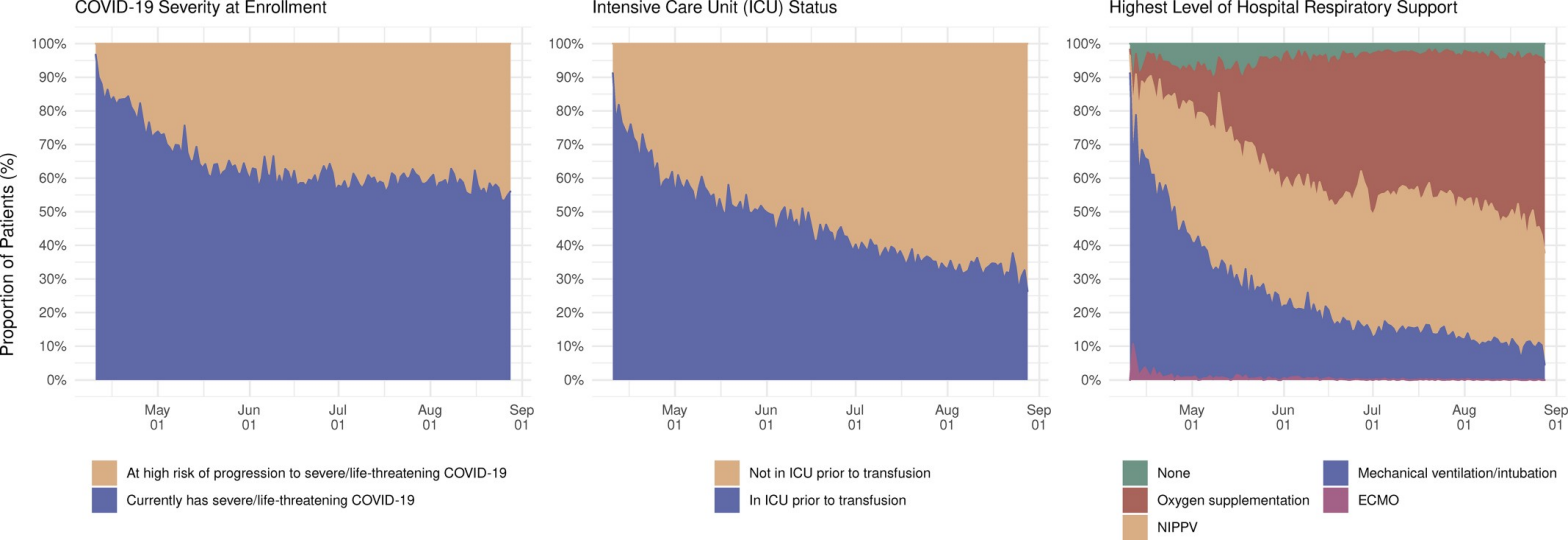
Daily patient enrollment in the US Expanded Access Program (EAP) relative to COVID-19 patient symptomatology. Stacked area chart displaying daily rates of patient enrollment in the EAP as a proportion of the sum total daily enrollment stratified by patient symptomatology, including 2 categories of COVID-19 disease severity, dichotomous representation of intensive care unit (ICU) status, and categorical level of respiratory support prior to COVID-19 convalescent plasma transfusion (none, oxygen supplementation, noninvasive positive-pressure ventilation [NIPPV], mechanical ventilation, or extracorporeal membrane oxygenation [ECMO]). Only patients who received a COVID-19 convalescent plasma transfusion are included in the 2 rightmost panels.

Enrollment in the EAP for convalescent plasma in each US state on each day of the EAP is displayed in Fig 4. The individual states and regional aggregates show clear patterns of when COVID-19 was surging during EAP enrollment. Fig 5 presents enrollment over time together with the number of active COVID-19 cases per US state. Chronologically, increases in enrollment in the EAP closely followed individual state infection rates. The number of patients enrolling in the EAP per 1,000 confirmed COVID-19 cases varied from 0.8 (Vermont) to 39.1 (Hawaii) across US states. The proportion that each US region contributed to total enrollment into the EAP varied throughout the program. Fig 6 displays proportional enrollment into the EAP across time by patient symptomatology, including COVID-19 disease severity at enrollment, ICU status, and level of respiratory support prior to COVID-19 convalescent plasma transfusion.

### Transfused patients

#### Transfused patient characteristics

Of the 105,717 patients enrolled in the EAP, about 90% of patients (94,287 patients) were transfused with a total of 112,654 units of COVID-19 convalescent plasma. As shown in Table 1, demographic and clinical characteristics at the time of transfusion were comparable between enrolled patients and transfused patients. Similarly, the subset of about 10% of patients (11,430 patients) who were enrolled in the EAP and not transfused with COVID-19 convalescent plasma was comparable to both enrolled patients and transfused patients.

#### Collection, distribution, and utilization of COVID-19 convalescent plasma

**Fig 7:**
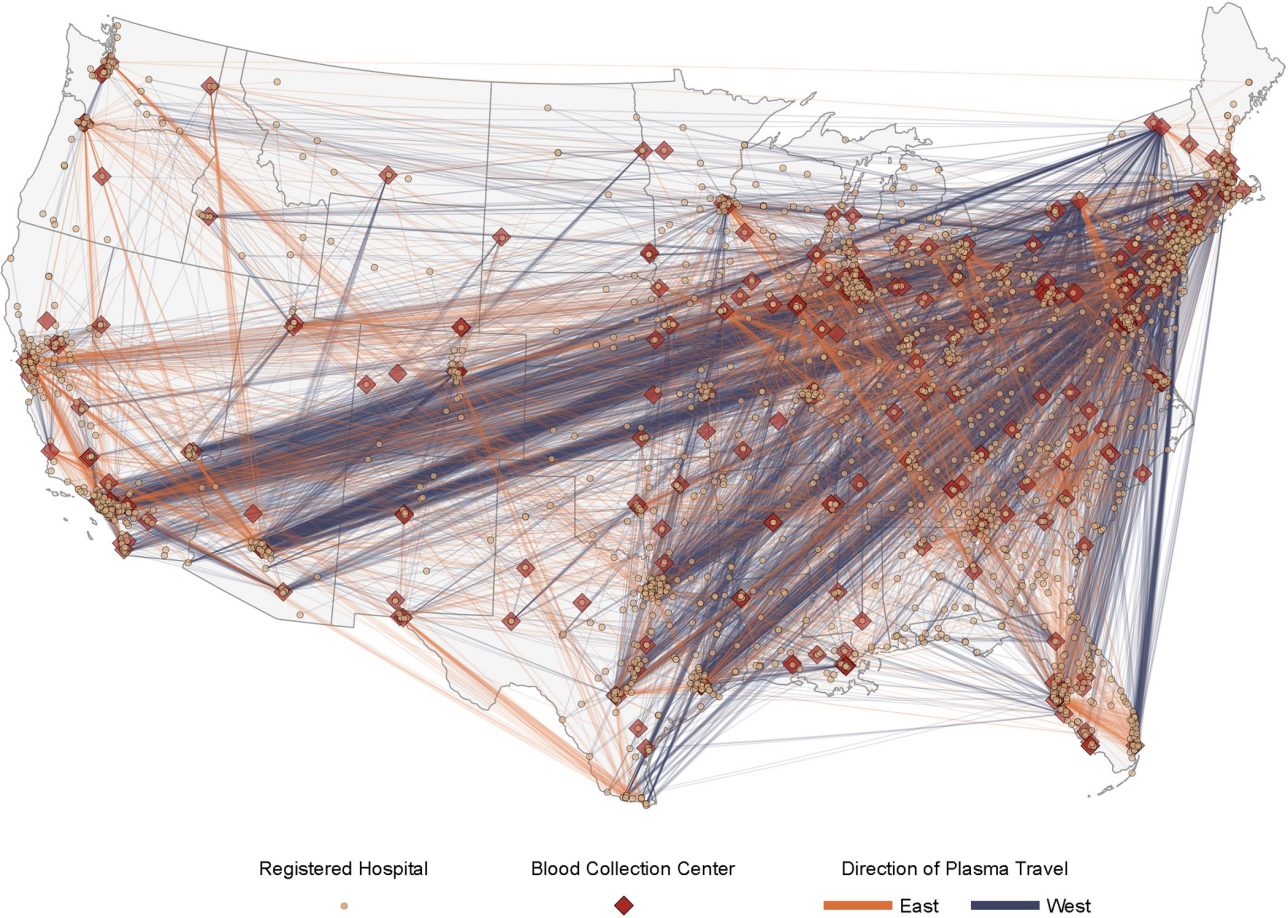
Travel paths of units of convalescent plasma from blood collection centers to sites of plasma transfusion within the contiguous US in the Expanded Access Program (EAP) for convalescent plasma. Map displaying the distance and direction of travel of convalescent plasma units in support of the EAP, with the thickness of each colored line directly proportional to the number of convalescent plasma units represented. Lines colored in blue represent a travel direction of east to west (e.g., New York City, NY, to Los Angeles, CA), and lines colored in orange represent a travel direction of west to east (e.g., Minneapolis, MN, to Tampa, FL). The US FDA–licensed or–registered blood collection facilities supplying plasma are presented as filled red diamonds, and acute care facilities are presented as filled yellow circles. The map does not display data from noncontiguous US locations, including facilities in Puerto Rico, Hawaii, and Alaska. The base layer of the geographical map was created using geographical data retrieved from the US Census Bureau (https://www2.census.gov/geo/tiger/TIGER2019/STATE/). No copyrighted material was used.

COVID-19 convalescent plasma was distributed by 313 individual collection facilities (as represented by facility identification numbers) that were situated over a broad geography in the US (Table 4), with collection centers in the South (35.8%), Midwest (25.6%), Northeast (18.5%), and West (18.5%). Given the geographical diversity of the participating blood collection centers, COVID-19 convalescent plasma was able to be transfused in close proximity to collection. The median distance between plasma collection or manufacturing center and transfusing hospital was 133 miles; 75% of all plasma units were transfused within 728 miles of the collection or manufacturing center. Resource sharing with parts of the country where collection sites were few resulted in approximately 20% of all plasma units traveling 1,000 miles or more prior to transfusion (Fig 7). Most of the overall COVID-19 convalescent plasma utilization was in the South (55.3%) and West (19.2%) geographical regions (Table 1; Fig 7). The states with the highest number of transfused patients were Texas (17,518), Florida (11,222), California (10,079), Georgia (4,919), New York (4,143), and Arizona (3,678) (Table 2).

**Table 4:** Summary of blood collection facilities, donations, and plasma distribution supporting the US Expanded Access Program (EAP) for convalescent plasma.

Measure	Number or number/total number (percent)
**Transfusions by the numbers**	
Patients transfused	94,287
Recorded transfusions	100,829
Total units given	112,654
Volume of plasma transfused (L)	25,223
Unique blood banks (FIN-based)	313
**Blood collection center region/territory**	
Midwest	80/313 (25.6%)
Northeast	58/313 (18.5%)
South	112/313 (35.8%)
West	58/313 (18.5%)
US territory	5/313 (1.6%)
**Travel distance of plasma donation to transfusion location (miles)**	
0 to 9	20,846/112,605 (18.5%)
10 to 49	20,399/112,605 (18.1%)
50 to 99	10,408/112,605 (9.2%)
99 to 149	6,599/112,605 (5.9%)
150 to 499	19,822/112,605 (17.6%)
500 to 999	11,483/112,605 (10.2%)
1,000 to 1,999	18,850/112,605 (16.7%)
2,000+	4,198/112,605 (3.7%)
**Units donated per region (subgrouped by receiving location)**	
Midwest	
Midwest	11,693/16,894 (69.2%)
South	3,176/16,894 (18.8%)
West	1,817/16,894 (10.8%)
Northeast	200/16,894 (1.2%)
US territory	8/16,894 (0.0%)
Northeast	
South	18,822/38,528 (48.9%)
Northeast	11,752/38,528 (30.5%)
West	4,175/38,528 (10.8%)
Midwest	3,713/38,528 (9.6%)
US territory	66/38,528 (0.2%)
South	
South	38,347/39,368 (97.4%)
Midwest	503/39,368 (1.3%)
Northeast	273/39,368 (0.7%)
West	236/39,368 (0.6%)
US territory	9/39,368 (0.0%)
West	
West	16,372/17,629 (92.9%)
South	776/17,629 (4.4%)
Midwest	380/17,629 (2.2%)
Northeast	97/17,629 (0.6%)
US territory	4/17,629 (0.0%)
US territory	
US territory	184/186 (98.9%)
South	2/186 (1.1%)

FIN, facility identification number.

#### Serious transfusion reactions

**Fig 8:**
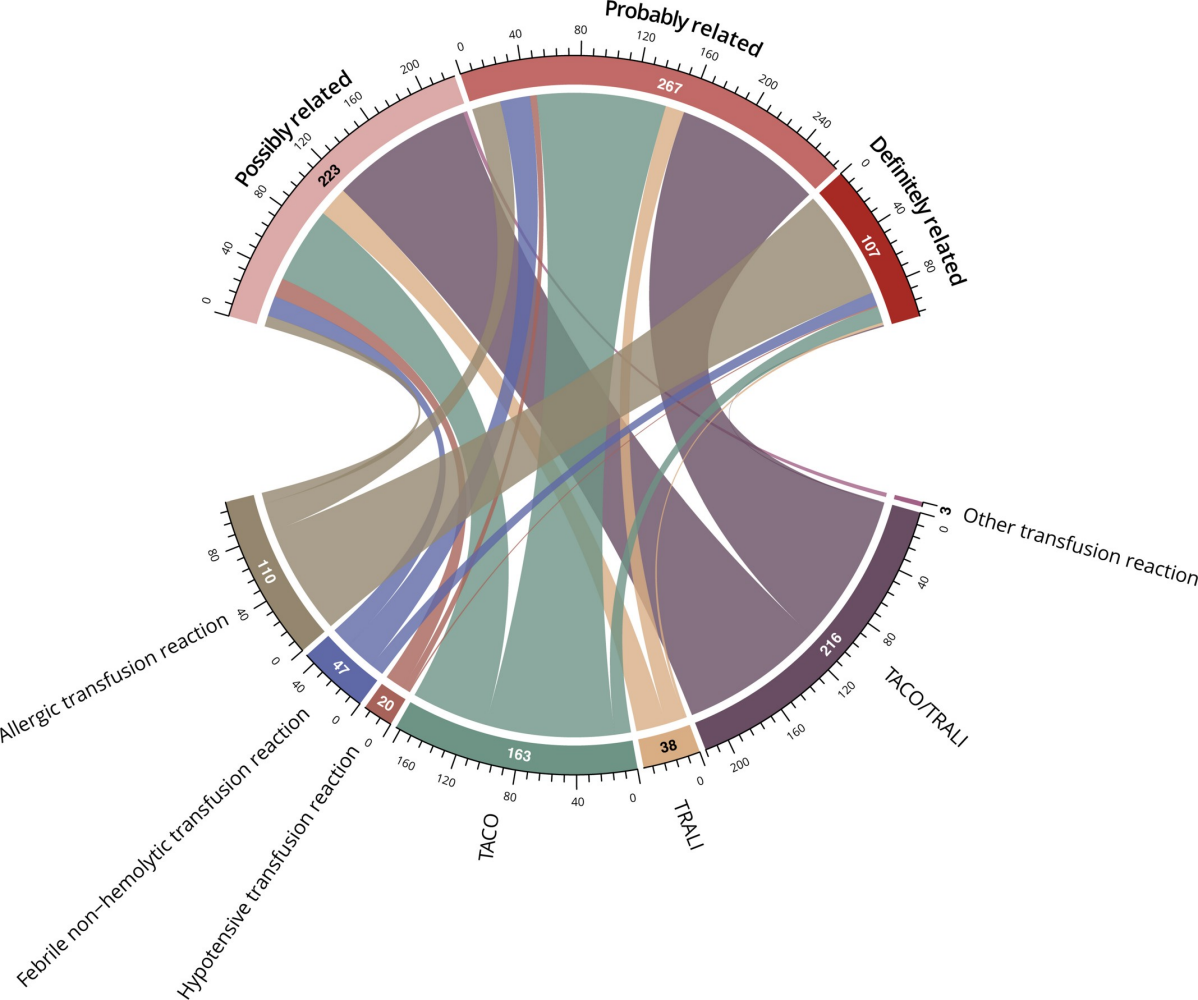
Chord diagram of the attributions associated with serious transfusion reactions. Attribution to a category of relatedness is depicted by a line connecting from each serious transfusion reaction type. The width of each line and the circumferential axis indicate the number of patients in each combined serious transfusion reaction and relatedness category group. Other transfusion reactions that were adjudicated to be possibly related to the transfusion (*n* = 3) included leukomoid reaction (*n* = 1), pulmonary embolism (*n* = 1), and red cell dilution (*n* = 1). TACO, transfusion-associated circulatory overload; TRALI, transfusion-related acute lung injury.

Key serious adverse events related to the transfusion of COVID-19 convalescent plasma are reported in Fig 8 and S2 Table. Our report is not a comprehensive summary of all risks associated with hospitalization for severe or life-threatening COVID-19. After adjudication, 597 serious adverse events were classified as related to convalescent plasma transfusion (<1% of all transfusions). TACO, TRALI, and severe allergic events were deemed “probably” or “definitely” related to convalescent plasma transfusion for most events, whereas hypotensive reactions were deemed “possibly” related in most cases. A broad grouping of TACO/TRALI was used to categorize events that initiated in close temporal proximity to the transfusion (within approximately 6 hours), with a broad clinical differential that most closely favored a diagnosis of TACO or TRALI.

#### Crude mortality among transfused patients

Mortality within 30 days of convalescent plasma transfusion was 25.2% (95% CI, 25.0% to 25.5%; Table 1). Crude mortality rates of transfused patients stratified by demographic characteristics, COVID-19 symptomatology, preexisting health conditions, concomitant medications, and other relevant clinical variables are displayed in Table 1. Crude mortality was 6.2% (95% CI, 5.3% to 7.3%) among transfused patients aged 20 to 29 years and 47.5% (95% CI, 45.3% to 49.7%) among patients aged 90 to 99 years. Crude mortality was 23.3% (95% CI, 22.9% to 23.7%) among females and 26.6% (95% CI, 26.2% to 27.0%) among males.

## Discussion

### Principal findings

The US EAP successfully provided access to COVID-19 convalescent plasma to over 105,000 patients, of whom nearly 95,000 patients were transfused with convalescent plasma over the course of 5 months. The EAP provided an efficient model for population-wide procurement, distribution, and infusion of convalescent plasma under a research protocol in a time of crisis. At its conclusion, the program was responsible for the largest number of transfusions of convalescent plasma for a single infectious disease to date. The EAP was initiated to respond to an emerging public health crisis. Both the scale and speed of execution of the program were notable: Over 25,000 hospitalized patients with COVID-19 were transfused with convalescent plasma within the first 11 weeks following the program’s inception. Access to convalescent plasma closely kept pace with increases in confirmed US COVID-19 infections per state over time, and there was substantial inclusion of vulnerable racial and ethnic minority populations. Geographically, enrollment in the EAP occurred in all US states, the District of Columbia, and the US territories of Puerto Rico and the US Virgin Islands. Patients were enrolled from all but 5 of the US national hospital referral regions, and substantial enrollment occurred in both metropolitan and non-metropolitan areas.

The rate of serious transfusion reactions was objectively low. The crude 30-day mortality rate in this high-risk patient population was 25.2%. Despite the potential risks associated with plasma transfusion in critically ill patients, these data provide evidence supporting the safety of COVID-19 convalescent plasma.

#### Demographic, chronological, and geographical characteristics of patients enrolled in the EAP

Demographic characteristics of the complete enrolled EAP cohort showed substantial enrollment among patients 60 years of age or older (57.8%), male patients (58.4%), patients of African American or Black race (18.2%), and patients of Hispanic or Latino ethnicity (37.2%). African American or Black race and Hispanic or Latino ethnicity make up 13.4% and 18.5% of the US population, respectively [30]. Previous studies have found that older [39,40], male [40], African American or Black race [39,41,42], and Hispanic or Latino [42] individuals are at higher risk of hospitalization for severe or life-threatening COVID-19. The presented enrollment results from the EAP highlight that this program was able to provide access to COVID-19 convalescent plasma to demographic groups that have experienced the largest disease burden from the US COVID-19 epidemic.

Participation in the EAP (both enrollments and transfusions) per day closely tracked the number of cases reported by state as the COVID-19 epidemic developed. Participation during April and May 2020 was high in Northeastern states, whereas in July and August participation peaked in the Southern region of mainland US, in line with the development of “hotspots” in these regions over time [43]. An increase in enrollment in Midwestern states was observed during August 2020, and this increase was also closely associated with an increase in confirmed COVID-19 cases in that region. Although there was widespread participation across US states and territories, there were fewer than 10 patients enrolled in both Vermont (*n* = 1) and Wyoming (*n* = 9), representing a small fraction of the total COVID-19 cases in those states. Given that there were no registered clinical trials involving COVID-19 convalescent plasma in these 2 states during the time of the EAP, there may have been regulatory or administrative barriers to participation in trials involving experimental therapeutics for COVID-19.

The EAP data reveal a gap between the number of enrolled and the number of transfused patients, particularly during the initial peak of each “wave” of the COVID-19 pandemic. This gap appears to represent the few days required to establish the local/regional infrastructure necessary to set up the collection and transfusion of convalescent plasma. Convalescent plasma was also shared between regions when the epidemic intensified in a certain region.

#### Strengths and limitations

The US EAP for COVID-19 convalescent plasma aimed to provide access to a treatment possibly providing benefit. Many of the trials of convalescent plasma experienced delays due to the unique challenges inherent to undertaking complex research during a public health crisis [44]. The EAP rapidly provided access to important information on the safety of COVID-19 convalescent plasma [23,24] while also providing signals of efficacy through exploratory analyses [22]. One of the federal requirements for an EAP is that it should not interfere with pivotal trials [45]. The EAP for COVID-19 convalescent plasma continuously transmitted data to the US FDA for ongoing evaluation of consistency with federal requirements for an EAP, and the program was not discontinued until the issuance of an EUA, which obviated the need for a convalescent plasma EAP.

Numerous challenges were encountered during the development and implementation of this national registry. In the context of competing demands on healthcare resources during the COVID19 pandemic [46], this national registry used a modern design with creative solutions to overcome the epidemiological and logistical challenges of the pandemic [47]. Creative solutions included a central academic IRB for oversight, streamlined registration for sites and physicians, simple online data collection forms, a robust support center that was accessible via email or telephone, minimal patient exclusion criteria, few restrictions on concomitant therapies, and no initiation or monitoring site visits. Several important limitations resulted from this design, however, including impact on data collection during “waves” (i.e., large increases in the number of cases of COVID-19 in the US) of the pandemic, unavailable data due to abridged data collection forms, and missing data due to the nature of a national registry. Additionally, the EAP was designed to provide access to convalescent plasma at hospitals and acute care facilities that were not already part of a clinical trial or did not have the infrastructure to support complex clinical trials. This registry also did not require training of the local investigators or study team members. This pragmatic approach did not ensure the highest quality of data nor completeness of data.

#### Implications for clinical practice and public policy

The success of the EAP in providing rapid access to convalescent plasma, combined with evidence supporting the safety of COVID-19 convalescent plasma, indicates that convalescent plasma is a “common sense” therapeutic that can be mobilized for future infectious disease outbreaks using the EAP methods as a model. In this regard it is noteworthy, given that COVID-19 convalescent plasma clinical trials were limited to only a few institutions, that most patients treated with plasma would have had no access without the EAP or single-patient eIND applications. The high use of convalescent plasma within the EAP indicates a high level of acceptance for this therapy by patients and frontline physicians despite the absence of high-quality data for its clinical efficacy. The EAP design was particularly effective in providing access to a potentially effective treatment in minority demographic groups and rural areas that are often underrepresented in US randomized controlled trials [48,49].

### Conclusion

The EAP provided rapid and broad access to convalescent plasma throughout the US and some US territories and was effective at providing therapy for demographic groups that were severely affected by COVID-19. Over time, the EAP provided access to convalescent plasma in response to sudden and exponential changes in SARS-CoV-2 infection rates. Data gathered from the EAP established that COVID-19 convalescent plasma was generally safe [23,24], and the EAP provided key efficacy data that were an important component of the scientific evidence considered by the US FDA in the decision to issue an EUA [22] for convalescent plasma in the treatment of hospitalized adults with COVID-19. Hence, this program established that it is possible to obtain relevant and actionable safety and efficacy data during pandemic conditions. The efficient study design of the EAP may serve as an example for future efforts when broad access to a treatment is needed in response to a rapidly developing infectious disease, providing access in areas typically underrepresented in clinical studies and thereby allowing capture of demographic groups that are often poorly represented in clinical trials.

## Supporting information

S1 Checklist STROBE checklist. (DOCX)

S1 Table Patient enrollment in the US Expanded Access Program (EAP) stratified by age, race, and ethnicity group, per 100,000 people (from the US census). (DOCX)

S2 Table Serious transfusion reaction characteristics in patients transfused with COVID-19 convalescent plasma. (DOCX)

S1 Text Study protocol and statistical analysis plan. (DOCX)

## Abbreviations

BARDA: Biomedical Advanced Research and Development Authority
COVID-19: coronavirus disease 2019
EAP: Expanded Access Program
eIND: emergency investigational new drug
EUA: emergency use authorization
FDA: Food and Drug Administration
ICU: intensive care unit
IND: investigational new drug
IRB: institutional review board
SARS-CoV-2: severe acute respiratory syndrome coronavirus 2
TACO: transfusion-associated circulatory overload
TRALI: transfusion-related acute lung injury
