# Supplementary material for: Access to and safety of COVID-19 convalescent plasma in the United States Expanded Access Program: A national registry study
Source: PLoS Med. 2021 Dec 20;18(12):e1003872. doi: 10.1371/journal.pmed.1003872 (PMC8730442; doi:10.1371/journal.pmed.1003872)
Supplement: S1 Checklist — (DOCX) [file pmed.1003872.s001.docx]

**S1 Checklist.** STROBE Checklist.

|  | | Item No | Recommendation | Section |
| --- | --- | --- | --- | --- |
| **Title and abstract** | | 1 | (***a***) Indicate the study’s design with a commonly used term in the title or the abstract | Title |
|  |  |  | (***b***) Provide in the abstract an informative and balanced summary of what was done and what was found | Abstract |
| Introduction | | | | |
| Background/rationale | | 2 | Explain the scientific background and rationale for the investigation being reported | Introduction 1^st^, 2^nd^, 3^rd^ paragraph |
| Objectives | | 3 | State specific objectives, including any prespecified hypotheses | Introduction 4^th^ paragraph |
| Methods | | | | |
| Study design | | 4 | Present key elements of study design early in the paper | Methods section first two paragraphs |
| Setting | | 5 | Describe the setting, locations, and relevant dates, including periods of recruitment, exposure, follow-up, and data collection | Methods 2.2, 2.4, 2.6 |
| Participants | | 6 | (***a***) Give the eligibility criteria, and the sources and methods of selection of participants. Describe methods of follow-up | Methods 2.1, 2.4, 2.6 |
|  |  |  | (***b***) For matched studies, give matching criteria and number of exposed and unexposed |  |
| Variables | | 7 | Clearly define all outcomes, exposures, predictors, potential confounders, and effect modifiers. Give diagnostic criteria, if applicable | Methods 2.4, 2.5, 2.6, 2.7 |
| Data sources/ measurement | | 8 | For each variable of interest, give sources of data and details of methods of assessment (measurement). Describe comparability of assessment methods if there is more than one group | Methods 2.3, 2.4, 2.5, 2.6, 2.7 |
| Bias | | 9 | Describe any efforts to address potential sources of bias | Methods 2.7 |
| Study size | | 10 | Explain how the study size was arrived at | Methods 2.4 |
| Quantitative variables | | 11 | Explain how quantitative variables were handled in the analyses. If applicable, describe which groupings were chosen and why | Methods 2.7 |
| Statistical methods | | 12 | (***a***) Describe all statistical methods, including those used to control for confounding | Methods 2.7 |
|  |  |  | (***b***) Describe any methods used to examine subgroups and interactions |  |
|  |  |  | (***c***) Explain how missing data were addressed |  |
|  |  |  | (***d***) If applicable, explain how loss to follow-up was addressed |  |
|  |  |  | (***e***) Describe any sensitivity analyses |  |
| Results | | | |  |
| Participants | | 13 | (**a**) Report numbers of individuals at each stage of study—eg numbers potentially eligible, examined for eligibility, confirmed eligible, included in the study, completing follow-up, and analysed | Results 3.1.1, 3.1.3, 3.2.1 |
|  |  |  | (**b**) Give reasons for non-participation at each stage |  |
|  |  |  | (**c**) Consider use of a flow diagram |  |
| Descriptive data | | 14 | (**a**) Give characteristics of study participants (eg demographic, clinical, social) and information on exposures and potential confounders | Results  3.1.1, 3.1.2, 3.1.3, 3.2.1, |
|  |  |  | (**b**) Indicate number of participants with missing data for each variable of interest | 3.2.2 |
|  |  |  | (**c**) Summarise follow-up time (eg, average and total amount) |  |
| Outcome data | | 15 | Report numbers of outcome events or summary measures over time | Results 3.1.1, 3.1.2, 3.1.3, 3.2.1, 3.2.2, 3.2.3., 3.2.4 |
| Main results | 16 | (***a***) Give unadjusted estimates and, if applicable, confounder-adjusted estimates and their precision (eg, 95% confidence interval). Make clear which confounders were adjusted for and why they were included | | Results 3.1.1, 3.1.2, 3.1.3, 3.2.1, 3.2.2, 3.2.3., 3.2.4 |
|  |  | (***b***) Report category boundaries when continuous variables were categorized | |  |
|  |  | (***c***) If relevant, consider translating estimates of relative risk into absolute risk for a meaningful time period | |  |
| Other analyses | 17 | Report other analyses done—eg analyses of subgroups and interactions, and sensitivity analyses | | N/A |
| Discussion | | | | |
| Key results | 18 | Summarise key results with reference to study objectives | | Discussion 4.1 |
| Limitations | 19 | Discuss limitations of the study, taking into account sources of potential bias or imprecision. Discuss both direction and magnitude of any potential bias | | Discussion 4.3 |
| Interpretation | 20 | Give a cautious overall interpretation of results considering objectives, limitations, multiplicity of analyses, results from similar studies, and other relevant evidence | | Discussion 4.2 |
| Generalisability | 21 | Discuss the generalisability (external validity) of the study results | | Discussion 4.4 |
| Other information | | | | |
| Funding | 22 | Give the source of funding and the role of the funders for the present study and, if applicable, for the original study on which the present article is based | | Funding/Support statement |
