# Supplementary material for: Access to and safety of COVID-19 convalescent plasma in the United States Expanded Access Program: A national registry study
Source: PLoS Med. 2021 Dec 20;18(12):e1003872. doi: 10.1371/journal.pmed.1003872 (PMC8730442; doi:10.1371/journal.pmed.1003872)
Supplement: S1 Table — (DOCX) [file pmed.1003872.s002.docx]

**S1 Table.** Patient enrollment in the US Expanded Access Program (EAP) stratified by race and ethnic group, relative to 100,000 people from US census per age, race, and ethnicity. This table provides summary data supporting **Figure 3**.

| **Race/Ethnicity and Age Grouping** | **Enrollments per 100,000 people from US census group** |
| --- | --- |
| **All races** |  |
| 20 - 29 | 5.91 |
| 30 - 39 | 15.55 |
| 40 - 49 | 32.56 |
| 50 - 59 | 50.88 |
| 60 - 69 | 70.30 |
| 70 - 79 | 95.20 |
| 80+ | 104.38 |
| **American Indian and Alaska Native alone** |  |
| 20 - 29 | 9.23 |
| 30 - 39 | 25.70 |
| 40 - 49 | 44.25 |
| 50 - 59 | 72.91 |
| 60 - 69 | 96.77 |
| 70 - 79 | 130.73 |
| 80+ | 125.87 |
| **Asian alone** |  |
| 20 - 29 | 2.70 |
| 30 - 39 | 6.33 |
| 40 - 49 | 12.74 |
| 50 - 59 | 30.22 |
| 60 - 69 | 48.68 |
| 70 - 79 | 68.41 |
| 80+ | 86.36 |
| **Black alone** |  |
| 20 - 29 | 6.79 |
| 30 - 39 | 21.34 |
| 40 - 49 | 46.11 |
| 50 - 59 | 78.16 |
| 60 - 69 | 124.70 |
| 70 - 79 | 174.03 |
| 80+ | 170.68 |
| **Native Hawaiian and Other Pacific Islander alone** |  |
| 20 - 29 | 22.17 |
| 30 - 39 | 48.71 |
| 40 - 49 | 113.69 |
| 50 - 59 | 171.10 |
| 60 - 69 | 239.97 |
| 70 - 79 | 271.81 |
| 80+ | 181.62 |
| **Two or more races** |  |
| 20 - 29 | 0.72 |
| 30 - 39 | 3.40 |
| 40 - 49 | 9.42 |
| 50 - 59 | 17.71 |
| 60 - 69 | 32.88 |
| 70 - 79 | 41.32 |
| 80+ | 44.83 |
| **White alone** |  |
| 20 - 29 | 3.54 |
| 30 - 39 | 8.83 |
| 40 - 49 | 18.97 |
| 50 - 59 | 31.06 |
| 60 - 69 | 45.49 |
| 70 - 79 | 69.26 |
| 80+ | 82.87 |
| **Hispanic** |  |
| 20 - 29 | 4.72 |
| 30 - 39 | 12.92 |
| 40 - 49 | 28.24 |
| 50 - 59 | 51.12 |
| 60 - 69 | 82.17 |
| 70 - 79 | 106.89 |
| 80+ | 104.08 |
