# Supplementary material for: Access to and safety of COVID-19 convalescent plasma in the United States Expanded Access Program: A national registry study
Source: PLoS Med. 2021 Dec 20;18(12):e1003872. doi: 10.1371/journal.pmed.1003872 (PMC8730442; doi:10.1371/journal.pmed.1003872)
Supplement: S2 Table — (DOCX) [file pmed.1003872.s003.docx]

**S2 Table.** Serious transfusion reaction characteristics in patients transfused with COVID-19 convalescent plasma. This table provides summary data supporting **Figure 8**.

| **SAE** | **Possibly related** | **Probably related** | **Definitely related** | **Total** |
| --- | --- | --- | --- | --- |
| Allergic transfusion reaction | 8 | 21 | 81 | **110** |
| Febrile non-hemolytic transfusion reaction | 15 | 22 | 10 | **47** |
| Hypotensive transfusion reaction | 14 | 5 | 1 | **20** |
| TACO | 56 | 95 | 12 | **163** |
| TRALI | 22 | 14 | 2 | **38** |
| TACO/TRALI | 105 | 110 | 1 | **216** |
| Other transfusion reaction | 3 | 0 | 0 | **3** |
| **Total** | **223** | **267** | **107** | **597** |
