# Supplementary material for: Access to and safety of COVID-19 convalescent plasma in the United States Expanded Access Program: A national registry study
Source: PLoS Med. 2021 Dec 20;18(12):e1003872. doi: 10.1371/journal.pmed.1003872 (PMC8730442; doi:10.1371/journal.pmed.1003872)
Supplement: S1 Text — (DOCX) [file pmed.1003872.s004.docx]

**S1 Text. Study Protocol and Statistical Analysis Plan.**

**This supplement contains the following items:**

1. **Study Protocol**
   1. Study Protocol (Version 2.0**^[[1]](#footnote-1)^**) **2**
   2. Study Protocol (Version 10.0) **21**
   3. Study Protocol Amendments Summary**^[[2]](#footnote-2)^** **44**
2. **Statistical Analysis Plan**
   1. Statistical Analysis Plan (Version 2.0**^[[3]](#footnote-3)^**) **47**
   2. Statistical Analysis Plan (Version 5.0) **50**
   3. Statistical Analysis Plan Summary of Changes **53**

**Study Protocol (version 2.0)**

Expanded Access to Convalescent Plasma for the Treatment of Patients with COVID-19

Unique Protocol Identification Number: 20-003312

National Clinical Trial (NCT) Identification Number: 04338360

Principal Investigator: Dr. Michael J. Joyner, MD

IND 19832 Sponsor: Dr. Michael J. Joyner, MD

Funded by: BARDA Contract No. 75A50120C00096

# Version 2.0

**03 April 2020**

The Mayo Clinic IRB will serve as the IRB of record for all sites participating in this protocol. In accordance with 45 CFR 46.103(e), agreeing to participate in the trial via sign up on [www.uscovidplasma.org](http://www.uscovidplasma.org) will serve as documentation of each participating institution’s reliance on Mayo’s IRB. A separate IRB reliance agreement is not required.

# Summary of Changes from Previous Version:

| **Affected Section(s)** | **Summary of Revisions Made** | **Rationale** |
| --- | --- | --- |
| **Cover Page** | Updated Version and added IRB of Record Statement | Clarification of IRB Oversight |
| **Synopsis** | Clarification of endpoints and duration of participation | For consistency throughout protocol |
| **8.3** | Changed reporting by treating physician to go to Sponsor | Direction from FDA for reporting requirements for INDs |

**Table of Contents**

1. [**Protocol Summary**](#_bookmark0)
   1. [Synopsis 5](#_bookmark1)
   2. [Schema 6](#_bookmark2)
2. [**Introduction** 7](#_bookmark3)
   1. [Study Rationale 7](#_bookmark4)
   2. [Background 7](#_bookmark5)
   3. [Risk/Benefit Assessment 7](#_bookmark6)
      1. [Known Potential Risks 7](#_bookmark7)
      2. [Known Potential Benefits 7](#_bookmark8)
      3. [Assessment of Potential Risks and Benefits 7](#_bookmark9)
3. [**Objectives and Endpoints** 8](#_bookmark10)
4. [**Study Design** 9](#_bookmark11)
   1. [Overall Design 9](#_bookmark12)
   2. [Scientific Rationale for Study Design 9](#_bookmark13)
   3. [Justification for Dose 9](#_bookmark14)
   4. [End of Study Definition 10](#_bookmark15)
5. [**Study Population** 1](#_bookmark16)1
   1. [Inclusion Criteria 11](#_bookmark17)
   2. [Strategies for Recruitment and Retention 11](#_bookmark18)
6. [**Study Intervention** 12](#_bookmark19)
   1. [Study Intervention(s) Administration 12](#_bookmark20)
      1. [Study Intervention Description 12](#_bookmark21)
      2. [Dosing and Administration 12](#_bookmark22)
   2. [Preparation/Handling/Storage/Accountability 12](#_bookmark23)
      1. [Acquisition and accountability 12](#_bookmark24)
      2. [Formulation, Appearance, Packaging, and Labeling 1](#_bookmark25)2
      3. [Product Storage and Stability 1](#_bookmark26)2
      4. [Preparation 1](#_bookmark27)2

[6.5 Concomitant Therapy 1](#_bookmark28)3

1. [**Study Intervention Discontinuation and Participant Discontinuation/Withdrawal** 1](#_bookmark29)4
   1. [Discontinuation of Study Intervention 1](#_bookmark30)4
   2. [Participant Discontinuation/Withdrawal from the Study 1](#_bookmark31)4
   3. [Lost to Follow-Up 1](#_bookmark32)4
2. [**Study Assessments and Procedures** 1](#_bookmark33)5
   1. [Efficacy Assessments 1](#_bookmark34)5
   2. [Safety and Other Assessments 1](#_bookmark35)5
   3. [Adverse Events and Serious Adverse Events 1](#_bookmark36)5
   4. [Unanticipated Problems 1](#_bookmark37)5
3. [**Statistical Considerations** 1](#_bookmark38)6
   1. [Statistical Hypotheses 1](#_bookmark39)6
   2. [Sample Size Determination 1](#_bookmark40)6
   3. [Populations for Analyses 1](#_bookmark41)6
   4. [Statistical Analyses 1](#_bookmark42)6
4. [**Supporting Documentation and Operational Considerations** 1](#_bookmark43)7
   1. [Regulatory, Ethical, and Study Oversight Considerations 1](#_bookmark44)7
      1. [Informed Consent Process 1](#_bookmark45)7
   2. [Abbreviations 1](#_bookmark46)9
   3. [Protocol Amendment History 1](#_bookmark47)9
5. [**References**](#_bookmark48) 20

[References](#_bookmark49) 20

# Statement of Compliance

The Expanded Access protocol will be carried out in accordance with applicable federal regulations:

o United States (US) Code of Federal Regulations (CFR) applicable to clinical studies (45 CFR Part 46, 21 CFR Part 50, 21 CFR Part 56, and 21 CFR Part 312).

# Treating Physician Responsibilities

The Sponsor may contact the treating physician as appropriate for completion of all data forms in a timely manner. You agree to respond to the sponsor in a timely manner with complete information. Failure to comply will be in violation with the protocol.

**1 PROTOCOL SUMMARY**

1.1 SYNOPSIS

| **Title:** | Expanded Access to Convalescent Plasma for the Treatment of Patients with COVID-19 |
| --- | --- |
| **Study Description:** | This expanded access program will provide access to investigational convalescent plasma for patients in acute care facilities infected with SARS-CoV-2 who have severe or life-threatening COVID-19, or who are judged by a healthcare provider to be at high risk of progression to severe or life-threatening disease. Following registration on the protocol and provision of informed consent, patients will be transfused with one unit of ABO compatible convalescent plasma obtained from an individual who has recovered from documented infection with SARS-CoV-2. Safety information collected will include serious adverse events judged to be related to the administration of convalescent plasma. Other information to be collected retrospectively will include patient demographics, acute care facility resource utilization (total length of stay, days in ICU, days intubated, and survival to discharge from an acute care facility. |
| **Objectives:** | Primary Objective: Provide access to COVID-19 convalescent plasma Secondary Safety  Objectives: |
| **Endpoints:** | Primary Endpoint: Availability of convalescent plasma Secondary Endpoints: Serious adverse events |
| **Study Population:** | Patients with severe or life-threatening manifestations of COVID-19, or documented to be at high risk of developing such manifestations |
| **Phase:** | Expanded Access Program |
| **Description of Sites/Facilities Enrolling Participants:** | Acute care facilities treating patients with COVID-19 |
| **Description of Study Intervention:** | Administration of convalescent plasma obtained from donors with prior documented SARS-CoV-2 infection |
| **Study Duration:** | 12 months |
| **Participant Duration:** | Patients will complete the study when they are discharged from the acute care facility in which the received the COVID-19 convalescent plasma. |

1.2 SCHEMA


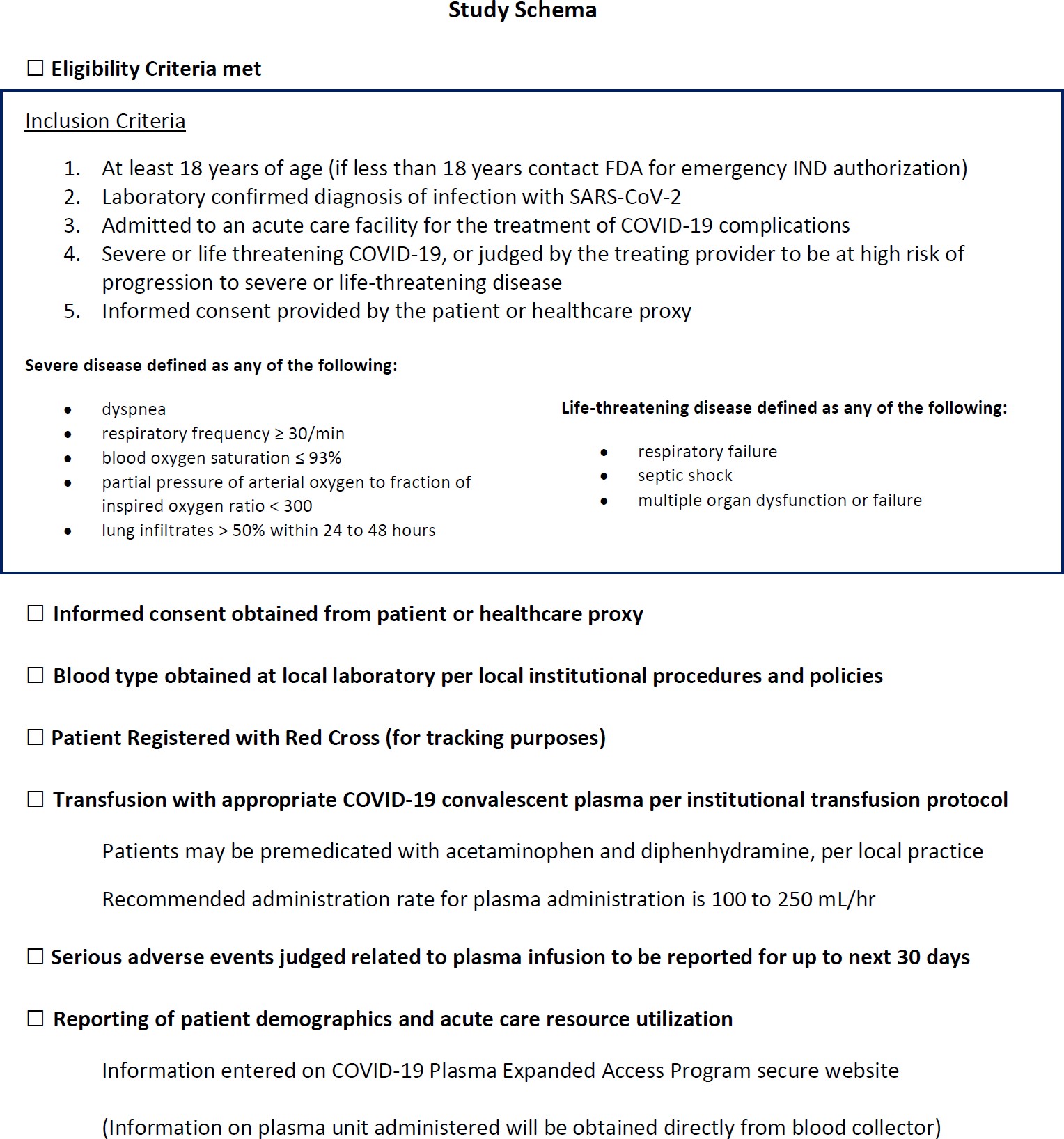


**by patient to provider**

**2 INTRODUCTION**

2.1 STUDY RATIONALE

Convalescent plasma has appeared to be of benefit for the treatment of certain infectious diseases, including infections from respiratory viruses. Preliminary evidence indicates that convalescent plasma may possibly be of benefit for some patients with COVID-19, leading to improvement.

2.2 BACKGROUND

One of the ways that people fight of infectious diseases is by developing antibodies that lead to the destruction of the invading microorganism. The antibodies are present in the blood, and more specifically in the liquid portion of the blood called plasma. People who have recovered from being recently infected can donate plasma and that plasma can then be given to individuals who are ill with the virus in order to try to help eliminate it from the system and allow them to get better. This has worked in previous outbreaks of respiratory diseases like influenza, and there are some early data to suggest that it might work for some people with COVID-19.^1,2^

2.3 RISK/BENEFIT ASSESSMENT

2.3.1 KNOWN POTENTIAL RISKS

Convalescent plasma represents a licensed blood product, for which the risks are well described. These

are the risks associated with the administration of plasma, including allergic reaction and viral infections. There is also the risk that convalescent plasma may be ineffective.

2.3.2 KNOWN POTENTIAL BENEFITS

COVID-19 convalescent plasma has not yet been demonstrated to provide clinical benefit in patients

affected by this disease.

2.3.3 ASSESSMENT OF POTENTIAL RISKS AND BENEFITS

Thee safety profile of plasma administration is well established. Taking into account the preliminary

data available on the possible benefit of COVID-19 plasma along with the relative lack of other readily available therapeutic options for severe or life-threatening disease, providing patients access to ABO compatible COVID-19 plasma as part of this expanded access protocol appears to reasonably balance potential risks with possible benefit that may outweigh such risks.

**3 OBJECTIVES AND ENDPOINTS**

| OBJECTIVES | ENDPOINTS | JUSTIFICATION FOR ENDPOINTS |
| --- | --- | --- |
| Primary | | |
| Provide access to COVID-19 convalescent plasma | Availability of convalescent plasma | Expanded access protocol |
| Secondary | | |
| Safety | Serious adverse events | Required as part of expanded access protocol under IND |
| Tertiary/Exploratory | | |
| Health care utilization | 1. Acute care facility length of stay 2. Days spent in intensive care unit 3. Survival to acute care facility discharge | Evaluation of potential for efficacy |

**4 STUDY DESIGN**

4.1 OVERALL DESIGN

This is an open-label expanded access program to make appropriately matched convalescent plasma available for the treatment of patients in acute care facilities infected with SARS-CoV-2 who have severe or life-threatening COVID-19, or who are judged by a healthcare provider to be at high risk of progression to severe or life-threatening disease. COVID-19 convalescent plasma will be obtained from blood suppliers and will meet all regulatory requirements for conventional plasma and FDA’s additional considerations for COVID-19 convalescent plasma ([https://www.fda.gov/vaccines-blood-](https://www.fda.gov/vaccines-blood-biologics/investigational-new-drug-ind-or-device-exemption-ide-process-cber/investigational-covid-19-convalescent-plasma-emergency-inds) [biologics/investigational-new-drug-ind-or-device-exemption-ide-process-cber/investigational-covid-19-](https://www.fda.gov/vaccines-blood-biologics/investigational-new-drug-ind-or-device-exemption-ide-process-cber/investigational-covid-19-convalescent-plasma-emergency-inds) [convalescent-plasma-emergency-inds](https://www.fda.gov/vaccines-blood-biologics/investigational-new-drug-ind-or-device-exemption-ide-process-cber/investigational-covid-19-convalescent-plasma-emergency-inds)). Information collected following plasma administration will include serious adverse events judged by the treating physician to be potentially related to the administration of the plasma, as well as patient demographics, acute care resource utilization, and characteristics of the convalescent plasma administered. Due to the nature of the COVID-19 outbreak, data on patient demographics, acute care resource utilization, and characteristics of the convalescent plasma administered may be collected retrospectively.

4.2 SCIENTIFIC RATIONALE FOR STUDY DESIGN

Convalescent plasma collected from individuals who have recovered from a prior viral infection for the passive transfer of antibodies has been used at various times over the past century. There has

been some evidence for benefit against hepatitis B, polio, measles, influenza, Ebola and other pathogens.^3^ Results from small case series during the prior MERS and SARS coronavirus outbreaks documented safety and faster viral clearance following convalescent plasma administration, particularly when given early in the disease course.^4,3^ Additionally, there is preliminary clinical evidence that suggests that convalescent plasma might provide benefit to individuals with SARS-CoV-2 infection and manifestations of COVID-19.^1,2^ At this time there are few therapeutic options for the treatment of COVID-19 and no prophylactic vaccine is currently available. Based on the preliminary evidence of possible efficacy, this protocol is making convalescent plasma available to individuals with documented SARS-CoV-2 disease at acute care facilities who have severe or life-threatening COVID-19, or who are judged by a provider to be at high risk of progression to severe or life-threatening disease.

4.3 JUSTIFICATION FOR DOSE

Initial data available from studies using COVID-19 convalescent plasma for the treatment of individuals with severe or life-threatening disease indicate that a single dose of 200 mL showed potential

efficacy. The volume of plasma to be transfused will be that of the full unit of COVID-19 convalescent plasma, or at least 200 mL.

4.4 END OF STUDY DEFINITION

Patients will complete the study when they are discharged from the acute care facility in which they received the COVID-19 convalescent plasma.

**5 STUDY POPULATION**

5.1 INCLUSION CRITERIA

1. Age at least 18 years
2. Laboratory confirmed diagnosis of infection with SARS-CoV-2
3. Admitted to an acute care facility for the treatment of COVID-19 complications
4. Severe or life threatening COVID-19, or judged by the treating provider to be at high risk of progression to severe or life-threatening disease
5. Informed consent provided by the patient or healthcare proxy

Severe COVID-19 is defined by one or more of the following:

- dyspnea
- respiratory frequency ≥ 30/min
- blood oxygen saturation ≤ 93%
- partial pressure of arterial oxygen to fraction of inspired oxygen ratio < 300
- lung infiltrates > 50% within 24 to 48 hours

Life-threatening COVID-19 is defined as one or more of the following:

- respiratory failure
- septic shock
- multiple organ dysfunction or failure

5.2 STRATEGIES FOR RECRUITMENT AND RETENTION

Patients eligible for this Expanded Access Program will be identified by their treating providers and

registered with the Red Cross. Since this involves the one-time administration of COVID-19 convalescent plasma to patients in acute care facilities, participant retention is not anticipated to be an issue.

**6 STUDY INTERVENTION**

6.1 STUDY INTERVENTION(S) ADMINISTRATION

This expanded program will make available ABO compatible COVID-19 convalescent plasma collected by

blood establishments in accordance with the AABB Circular of Information and FDA's regulations and the additional considerations for convalescent plasma on FDA’s webpage ([https://www.fda.gov/vaccines-](https://www.fda.gov/vaccines-blood-biologics/investigational-new-drug-ind-or-device-exemption-ide-process-cber/investigational-covid-19-convalescent-plasma-emergency-inds) [blood-biologics/investigational-new-drug-ind-or-device-exemption-ide-process-cber/investigational-](https://www.fda.gov/vaccines-blood-biologics/investigational-new-drug-ind-or-device-exemption-ide-process-cber/investigational-covid-19-convalescent-plasma-emergency-inds) [covid-19-convalescent-plasma-emergency-inds](https://www.fda.gov/vaccines-blood-biologics/investigational-new-drug-ind-or-device-exemption-ide-process-cber/investigational-covid-19-convalescent-plasma-emergency-inds)).^5^

6.1.1 STUDY INTERVENTION DESCRIPTION

ABO compatible COVID-19 convalescent plasma will be administered according to standard hospital

procedures.

6.1.2 DOSING AND ADMINISTRATION

For practical purposes in the current outbreak, one unit of ABO compatible COVID-19 convalescent

plasma will be administered. This will be provided by a registered or licensed blood collector and will be collected preferably by apheresis (volume of plasma to administer approximately 200-400 mL), or if necessary, by conventional methods (volume of plasma to administer approximately 200-250 mL).

Individual institutional guidelines for the administration of plasma should be followed, including the use of any premedications, such as acetaminophen and diphenhydramine. The duration of infusion will usually take 1 to 2 hours (rate of 100 to 250 mL/hr).

6.2 PREPARATION/HANDLING/STORAGE/ACCOUNTABILITY

6.2.1 ACQUISITION AND ACCOUNTABILITY

ABO compatible convalescent plasma units may be obtained from a registered or licensed blood

collector following registration of the patient.

6.2.2 FORMULATION, APPEARANCE, PACKAGING, AND LABELING

COVID-19 convalescent plasma will be supplied as an investigational blood product for the treatment of

COVID-19 with either a label or tie tag on the bag indicating the presence of COVID-19 antibodies.

6.2.3 PRODUCT STORAGE AND STABILITY

Please see the AABB Circular of Information for product storage and stability.

6.2.4 PREPARATION

Please follow local institutional guidelines for conventional plasma administration for COVID-19 plasma

preparation prior to administration, including ABO compatibility checks and thawing.

6.5 CONCOMITANT THERAPY

Premedications may be administered prior to plasma administration according to individual acute care

facility protocols.

**7 STUDY INTERVENTION DISCONTINUATION AND PARTICIPANT DISCONTINUATION/WITHDRAWAL**

7.1 DISCONTINUATION OF STUDY INTERVENTION

This study involves the one-time administration of ABO matched COVID-19 convalescent

plasma. Patients are free to withdraw consent from participation at any time.

7.2 PARTICIPANT DISCONTINUATION/WITHDRAWAL FROM THE STUDY

Patient are free to withdraw consent from participation in further data collection at any time during the

study.

7.3 LOST TO FOLLOW-UP

Since this protocol involves only the acute care of individuals with COVID-19, it is not anticipated that

individuals will be lost to follow-up.

**8 STUDY ASSESSMENTS AND PROCEDURES**

8.1 EFFICACY ASSESSMENTS

Patient demographic information will be obtained to include age and sex

Assessments for potential efficacy are exploratory as part of this expanded access program but may include web-based collection of the following information:

1. Acute care facility length of stay
2. Number of days in an intensive care unit
3. Number of days on mechanical ventilation
4. Survival until discharge from an acute care facility

An exploratory analysis may be conducted correlating the level of neutralizing antibody titers with clinical outcomes observed.

8.2 SAFETY AND OTHER ASSESSMENTS

Reporting will only be required for serious adverse events judged by the treating physician to be potentially related to the administration of COVID-19 convalescent plasma.

8.3 ADVERSE EVENTS AND SERIOUS ADVERSE EVENTS

Serious adverse events judged by the treating physician to be potentially related to the administration of COVID-19 convalescent plasma should be reported to the sponsor/principal investigator. Sponsor reports periodically to FDA.

8.4 UNANTICIPATED PROBLEMS

Any unanticipated issues should be reported to the principal investigator and to the IND Sponsor,

Michael Joyner.

**9 STATISTICAL CONSIDERATIONS**

9.1 STATISTICAL HYPOTHESES

This is an expanded access program and any statistical analyses for safety or efficacy will be exploratory.

9.2 SAMPLE SIZE DETERMINATION

This is an expanded access protocol that is intended to supply ABO compatible COVID-19 convalescent plasma to individuals with severe or life-threatening disease or at high risk thereof. It is anticipated that up to several thousand COVID-19 patients might be enrolled, though this number is difficult to estimate, given the evolving nature of the current pandemic.

9.3 POPULATIONS FOR ANALYSES

Serious adverse events will be collected on all treated individuals.

Exploratory analyses will be performed on data obtained from individuals providing informed consent.

9.4 STATISTICAL ANALYSES

Only exploratory statistical analyses will be performed as part of this expanded access program.

**10 SUPPORTING DOCUMENTATION AND OPERATIONAL CONSIDERATIONS**

10.1 REGULATORY, ETHICAL, AND STUDY OVERSIGHT CONSIDERATIONS

Plasma represents a licensed blood product. However, the use of plasma for the treatment of COVID-19

is investigational.

10.1.1 INFORMED CONSENT PROCESS

Since the use of COVID-19 plasma is investigational at this time, a discussion of potential risks and

benefits to its administration, as well as alternative options, should take place with patients or their healthcare proxies. This should be documented in the medical record.

10.1.1.1 CONSENT/ASSENT AND OTHER INFORMATIONAL DOCUMENTS PROVIDED TO PARTICIPANTS

Below is a template of language that can be used for informed consent. The language has been

deliberately streamlined for use in the setting of the current COVID-19 pandemic.

10.1.1.2 CONSENT PROCEDURES AND DOCUMENTATION

Consent must be obtained from the patient or the patient's health care proxy prior to treatment.

**Example of a consent form for treatment with experimental convalescent plasma therapy follows. IRB Reliance**

**Your request to participate in the EAP, use of the product and the consent form indicates your willingness and that of your institution/hospital/practice/legal business entity to rely upon the Mayo Clinic IRB and that you will follow all federal and state regulations regarding use and administration of the investigational product and that you will conduct the EAP in accordance with the principles set forth in the Belmont Report.**

**Safety Oversight**

**The Mayo Clinic IRB, a DSMB and the US FDA will work collaboratively to follow SAE reporting and monitor the conduct of the EAP.**

**The DSMB will be composed of experienced physicians and scientists who understand the risks of administered products and have sufficient research and trial experience to provide an independent recommendation to the PI, the IRB and the FDA. The FDA will hold the ultimate decision-making power to terminate the study evidence of early benefit or harm. The DSMB will generate monthly reports to the IRB and FDA, and the DSMB Chair, the PI, the appropriate FDA officials will review SAE aggregates weekly or less often as appropriate to ensure appropriate safety oversight.**

**DSMB Membership**

**The DSMB membership will include:**

**Allan S. Jaffe, MD, - Chair**

**Professor of Medicine and Laboratory Medicine**

**Consultant in the Division of Cardiac Critical Care and Ischemic Heart Disease Mayo Clinic, Rochester, MN**

**David O. Warner, MD - Secretary Professor ofAnesthesiology**

**Mayo Clinic, Rochester, MN**

**William Morice, MD**

**Professor of Laboratory Medicine**

**Chair, Department of Laboratory Medicine and Pathology Mayo Clinic, Rochester, MN**

**Paula Santrach, MD**

**Associate Professor of Laboratory Medicine and Pathology Consultant in Transfusion Medicine**

**Mayo Clinic, Rochester, MN**

**Robert L. Frye, MD**

**Professor of Medicine, Mayo Clinic and Past Chair, Department of Medicine Mayo Clinic, Rochester, MN**

**Ex Officio**

**Taimur Sher, MD, Associate Professor of Medicine, Co-Chair Mayo Clinic Thursday IRB**

**Consultant in the Division of Hematology Mayo Clinic, Jacksonville, FL**

10.2 ABBREVIATIONS

| AE | Adverse Event |
| --- | --- |
| CFR | Code of Federal Regulations |
| CRF | Case Report Form |
| EC | Ethics Committee |
| eCRF | Electronic Case Report Forms |
| FDA | Food and Drug Administration |
| IB | Investigator’s Brochure |
| IND | Investigational New Drug Application |
| IRB | Institutional Review Board |
| NIH | National Institutes of Health |
| SAE | Serious Adverse Event |
| SAP | Statistical Analysis Plan |
| SOP | Standard Operating Procedure |
| US | United States |

| 10.3 PROTOCOL AMENDMENT HISTORY | | | | |
| --- | --- | --- | --- | --- |
| **Version** | **Date** | **Description of Change** | **Brief Rationale** |  |
|  |  |  |  |  |
|  |  |  |  |  |
|  |  |  |  |  |
|  |  |  |  |  |
|  |  |  |  |  |
|  |  |  |  |  |
|  |  |  |  |  |
|  |  |  |  |  |

**11 REFERENCES**

REFERENCES

1. Roback JD, Guarner J, Convalescent Plasma to Treat COVID-19: Possibilities and Challenges., JAMA. 2020 Mar 27;

[Pmid:32219429](https://www.ncbi.nlm.nih.gov/PubMed/32219429)

1. Shen C, Wang Z, Zhao F, Yang Y, Li J, Yuan J, Wang F, Li D, Yang M, Xing L, Wei J, Xiao H, Yang Y, Qu J, Qing L, Chen L, Xu Z, Peng L, Li Y, Zheng H, Chen F, Huang K, Jiang Y, Liu D, Zhang Z, Liu Y, Liu L, Treatment of 5 Critically Ill Patients With COVID-19 With Convalescent Plasma., JAMA. 2020 Mar 27;

[Pmid:32219428](https://www.ncbi.nlm.nih.gov/PubMed/32219428)

1. Mair-Jenkins J, Saavedra-Campos M, Baillie JK, Cleary P, Khaw FM, Lim WS, Makki S, Rooney KD, Nguyen- Van-Tam JS, Beck CR, Convalescent Plasma Study Group., The effectiveness of convalescent plasma and hyperimmune immunoglobulin for the treatment of severe acute respiratory infections of viral etiology: a systematic review and exploratory meta-analysis., J Infect Dis. 2015 Jan 1;211(1):80-90

[Pmid:25030060](https://www.ncbi.nlm.nih.gov/PubMed/25030060)

1. Ko JH, Seok H, Cho SY, Ha YE, Baek JY, Kim SH, Kim YJ, Park JK, Chung CR, Kang ES, Cho D, Müller MA, Drosten C, Kang CI, Chung DR, Song JH, Peck KR, Challenges of convalescent plasma infusion therapy in Middle East respiratory coronavirus infection: a single centre experience., Antivir Ther. 2018;23(7):617- 622

[Pmid:29923831](https://www.ncbi.nlm.nih.gov/PubMed/29923831)

1. <http://www.aabb.org/tm/coi/Pages/default.aspx>

**Study Protocol (version 10.0)**

**Expanded Access to Convalescent Plasma for the Treatment of Patients with COVID-19**

**Unique Protocol Identification Number: 20-003312**

**National Clinical Trial (NCT) Identification Number: 04338360**

**Principal Investigator: Dr. Michael J. Joyner, MD**

**IND 19832 Sponsor: Dr. Michael J. Joyner, MD**

**Funded by: BARDA Contract No. 75A50120C00096**

**Version 10.0**

**03 August 2020**

The Mayo Clinic IRB will serve as the IRB of record for all sites participating in this protocol. In accordance with 45 CFR 46.103(e), agreeing to participate in the trial via sign up on [www.uscovidplasma.org](http://www.uscovidplasma.org) will serve as documentation of each participating institution’s reliance on Mayo’s IRB. A separate IRB reliance agreement is not required.

**Summary of Changes from Previous Version:**

| **Affected Section(s)** | **Summary of Revisions Made** | **Rationale** |
| --- | --- | --- |
| **1.2 and 5.1** | Eligibility criteria - Clinically suspected diagnosis should include a pending laboratory test result. | Patients enrolled based on clinically suspected infection with SARS-CoV-2 should have pending laboratory confirmation. |

**Table of Contents**

1. [**Protocol Summary** 24](#_bookmark0)
   1. [Synopsis 24](#_bookmark1)
   2. [Schema 25](#_bookmark2)
2. [**Introduction** 26](#_bookmark3)
   1. [Study Rationale 26](#_bookmark4)
   2. [Background 26](#_bookmark5)
   3. [Risk/Benefit Assessment 26](#_bookmark6)
      1. [Known Potential Risks 26](#_bookmark7)
      2. [Known Potential Benefits 26](#_bookmark8)
      3. [Assessment of Potential Risks and Benefits 26](#_bookmark9)
3. [**Objectives and Endpoints** 27](#_bookmark10)
4. [**Study Design** 28](#_bookmark11)
   1. [Overall Design 28](#_bookmark12)
   2. [Scientific Rationale for Study Design 28](#_bookmark13)
   3. [Justification for Dose 29](#_bookmark14)
   4. [Convalescent Plasma Compatibility 29](#_bookmark15)
   5. [End of Study Definition 29](#_bookmark16)
5. [**Study Population** 30](#_bookmark17)
   1. [Inclusion Criteria 30](#_bookmark18)
   2. [Strategies for Recruitment and Retention 30](#_bookmark19)
6. [**Study Intervention** 31](#_bookmark20)
   1. [Study Intervention(s) Administration 31](#_bookmark21)
      1. [Study Intervention Description 31](#_bookmark22)
      2. [Dosing and Administration 31](#_bookmark23)
   2. [Preparation/Handling/Storage/Accountability 31](#_bookmark24)
      1. [Acquisition and accountability 31](#_bookmark25)
      2. [Formulation, Appearance, Packaging, and Labeling 31](#_bookmark26)
      3. [Product Storage and Stability 32](#_bookmark27)
      4. [Preparation 32](#_bookmark28)
   3. [Concomitant Therapy 32](#_bookmark29)
7. [**Study Intervention Discontinuation and Participant Discontinuation/Withdrawal** 33](#_bookmark30)
   1. [Discontinuation of Study Intervention 33](#_bookmark31)
   2. [Participant Discontinuation/Withdrawal from the Study 33](#_bookmark32)
   3. [Lost to Follow-Up 33](#_bookmark33)
8. [**Study Assessments and Procedures** 34](#_bookmark34)
   1. [Efficacy Assessments 34](#_bookmark35)
   2. [Safety and Other Assessments 34](#_bookmark36)
   3. [Adverse Events and Serious Adverse Events 34](#_bookmark37)
   4. [Unanticipated Problems 34](#_bookmark38)
9. [**Statistical Considerations** 35](#_bookmark39)
   1. [Statistical Hypotheses 35](#_bookmark40)
   2. [Sample Size Determination 35](#_bookmark41)
   3. [Populations for Analyses 35](#_bookmark42)
   4. [Statistical Analyses 35](#_bookmark43)
   5. [Publication Plan 35](#_bookmark44)
10. [**Supporting Documentation and Operational Considerations** 36](#_bookmark45)
    1. [Regulatory, Ethical, and Study Oversight Considerations 36](#_bookmark46)
       1. [Informed Consent Process 36](#_bookmark47)
    2. [IRB Reliance 37](#_bookmark48)
    3. [Safety Oversight 37](#_bookmark49)
    4. [Abbreviations 39](#_bookmark50)
    5. [Protocol Amendment History 40](#_bookmark51)
11. [**References** 43](#_bookmark52)

[References 43](#_bookmark53)

# Statement of Compliance

The Expanded Access protocol will be carried out in accordance with applicable federal regulations:

o United States (US) Code of Federal Regulations (CFR) applicable to clinical studies (45 CFR Part 46, 21 CFR Part 50, 21 CFR Part 56, and 21 CFR Part 312).

# Treating Physician Responsibilities

The Sponsor may contact the treating physician as appropriate for completion of all data forms in a timely manner. You agree to respond to the sponsor in a timely manner with complete information. Failure to comply will be in violation with the protocol.

**1 PROTOCOL SUMMARY**

1.1 SYNOPSIS

| **Title:** | Expanded Access to Convalescent Plasma for the Treatment of Patients with COVID-19 |
| --- | --- |
| **Study Description:** | This expanded access program will provide access to investigational convalescent plasma for patients in acute care facilities infected with SARS-CoV-2 who have severe or life-threatening COVID-19, or who are judged by a healthcare provider to be at high risk of progression to severe or life-threatening disease. Following registration on the protocol and provision of informed consent, patients will be transfused with one (or more) units of convalescent plasma obtained from an individual who has recovered from documented infection with SARS-CoV-2. Safety information collected will include serious adverse events. Other information to be collected retrospectively will include patient demographics, acute care facility resource utilization (total length of stay, days in ICU, days intubated, and survival to discharge from an acute care facility. |
| **Objectives:** | Primary Objective: Provide access to COVID-19 convalescent plasma Secondary Safety  Objectives: |
| **Endpoints:** | Primary Endpoint: Availability of convalescent plasma Secondary Endpoints: Serious adverse events |
| **Study Population:** | Patients with severe or life-threatening manifestations of COVID-19, or documented to be at high risk of developing such manifestations |
| **Phase:** | Expanded Access Program |
| **Description of Sites/Facilities Enrolling Participants:** | Acute care facilities treating patients with COVID-19 |
| **Description of Study Intervention:** | Administration of convalescent plasma obtained from donors with prior documented SARS-CoV-2 infection |
| **Study Duration:** | 12 months |
| **Participant Duration:** | Patients will complete the study when they are discharged from the acute care facility in which the received the COVID-19 convalescent plasma. |

1.2 SCHEMA

**Study Schema**

Eligibility Criteria Met

Inclusion Criteria

1. At least 18 years of age (if less than 18 years contact FDA for emergency IND authorization)
2. Laboratory confirmed or clinically suspected diagnosis of infection with SARS-CoV-2*
3. Admitted to an acute care facility for the treatment of COVID-19 complications
4. Severe or life threatening COVID-19, or judged by the treating provider to be at high risk of progression to severe or life-threatening disease
5. Informed consent provided by the patient or healthcare proxy

*Patients enrolled based on clinically suspected infection with SARS-CoV-2 should have pending laboratory

confirmation.

| **Severe disease defined as any of the following:** | **Life-threatening disease defined as any of the following:** |
| --- | --- |
| - dyspnea - respiratory frequency> 30/min - blood oxygen saturation ≤ 93% - partial pressure of arterial oxygen to fraction of inspired oxygen ratio < 300 - lung infiltrates > 50% within 24 to 48 hours | - respiratory failure - septic shock - multiple organ dysfunction or failure |

Informed consent obtained from patient or healthcare proxy

Blood type obtained at local laboratory per local institutional procedures and policies Patient Registered with American Red Cross or other blood source by uscovidplasma.org.

Transfusion with appropriate COVID-19 convalescent plasma per institutional transfusion protocol

Patients may be premedicated with acetaminophen and diphenhydramine, per local practice

Recommended administration rate for plasma administration should follow best clinical practice procedures for the patient’s unique condition and your facility guidelines. r

Serious adverse events observed by the treating physician or reported by patient to provider Reporting of patient demographics and acute care resource utilization

Information entered on COVID-19 Plasma Expanded Access Program secure website (Information on plasma unit administered will be obtained directly from blood collector)

**2 INTRODUCTION**

2.1 STUDY RATIONALE

Convalescent plasma has appeared to be of benefit for the treatment of certain infectious diseases, including infections from respiratory viruses. Preliminary evidence indicates that convalescent plasma may possibly be of benefit for some patients with COVID-19, leading to improvement.

2.2 BACKGROUND

One of the ways that people fight of infectious diseases is by developing antibodies that lead to the destruction of the invading microorganism. The antibodies are present in the blood, and more specifically in the liquid portion of the blood called plasma. People who have recovered from being recently infected can donate plasma and that plasma can then be given to individuals who are ill with the virus in order to try to help eliminate it from the system and allow them to get better. This has worked in previous outbreaks of respiratory diseases like influenza, and there are some early data to suggest that it might work for some people with COVID-19.^1,2^

2.3 RISK/BENEFIT ASSESSMENT

2.3.1 KNOWN POTENTIAL RISKS

Plasma represents a licensed blood product, for which the risks are well described. These are the risks

associated with the administration of plasma, including allergic reaction and viral infections. There is also the risk that convalescent plasma may be ineffective.

2.3.2 KNOWN POTENTIAL BENEFITS

COVID-19 convalescent plasma has not yet been demonstrated to provide clinical benefit in patients

affected by this disease.

2.3.3 ASSESSMENT OF POTENTIAL RISKS AND BENEFITS

The safety profile of plasma administration is well established. Taking into account the preliminary data

available on the possible benefit of COVID-19 plasma along with the relative lack of other readily available therapeutic options for severe or life-threatening disease, providing patients access to compatible COVID-19 plasma as part of this expanded access protocol appears to reasonably balance potential risks with possible benefit that may outweigh such risks.

**3 OBJECTIVES AND ENDPOINTS**

| OBJECTIVES | ENDPOINTS | JUSTIFICATION FOR ENDPOINTS |
| --- | --- | --- |
| Primary | | |
| Provide access to COVID-19 convalescent plasma | Availability of convalescent plasma | Expanded access protocol |
| Secondary | | |
| Safety | Serious adverse events | Required as part of expanded access protocol under IND |
| Tertiary/Exploratory | | |
| Health care utilization | 1. Acute care facility length of stay 2. Days spent in intensive care unit 3. Survival to acute care facility discharge | Evaluation of potential for efficacy |

**4 STUDY DESIGN**

4.1 OVERALL DESIGN

This is an open-label expanded access program to make appropriately matched convalescent plasma available for the treatment of patients in acute care facilities infected with SARS-CoV-2 who have severe or life-threatening COVID-19, or who are judged by a healthcare provider to be at high risk of progression to severe or life-threatening disease. COVID-19 convalescent plasma will be obtained from blood suppliers and will meet all regulatory requirements for conventional plasma and FDA’s additional considerations for COVID-19 convalescent plasma ([https://www.fda.gov/vaccines-blood-](https://www.fda.gov/vaccines-blood-biologics/investigational-new-drug-ind-or-device-exemption-ide-process-cber/investigational-covid-19-convalescent-plasma-emergency-inds) [biologics/investigational-new-drug-ind-or-device-exemption-ide-process-cber/investigational-covid-19-](https://www.fda.gov/vaccines-blood-biologics/investigational-new-drug-ind-or-device-exemption-ide-process-cber/investigational-covid-19-convalescent-plasma-emergency-inds) [convalescent-plasma-emergency-inds](https://www.fda.gov/vaccines-blood-biologics/investigational-new-drug-ind-or-device-exemption-ide-process-cber/investigational-covid-19-convalescent-plasma-emergency-inds)). Information collected following plasma administration will include serious adverse events, as well as patient demographics, acute care resource utilization, and characteristics of the convalescent plasma administered. Due to the nature of the COVID-19 outbreak, data on patient demographics, acute care resource utilization, and characteristics of the convalescent plasma administered may be collected retrospectively.

4.2 SCIENTIFIC RATIONALE FOR STUDY DESIGN

Convalescent plasma collected from individuals who have recovered from a prior viral infection for the passive transfer of antibodies has been used at various times over the past century. There has

been some evidence for benefit against hepatitis B, polio, measles, influenza, Ebola and other pathogens.^3^ Results from small case series during the prior MERS and SARS coronavirus outbreaks documented safety and faster viral clearance following convalescent plasma administration, particularly when given early in the disease course.^4,3^ Additionally, there is preliminary clinical evidence that suggests that convalescent plasma might provide benefit to individuals with SARS-CoV-2 infection and manifestations of COVID-19.^1,2^ At this time there are few therapeutic options for the treatment of COVID-19 and no prophylactic vaccine is currently available. Based on the preliminary evidence of possible efficacy, this protocol is making convalescent plasma available to individuals with documented SARS-CoV-2 disease at acute care facilities who have severe or life-threatening COVID-19, or who are judged by a provider to be at high risk of progression to severe or life-threatening disease.

4.3 JUSTIFICATION FOR DOSE

Initial data available from studies using COVID-19 convalescent plasma for the treatment of individuals with severe or life-threatening disease indicate that a single dose of 200 mL showed potential efficacy. The volume of plasma to be transfused should be based upon the patient’s weight and clinical comorbidities (e.g. patients with impaired cardiac function and heart failure may require less volume or more prolonged transfusion times). The volume of plasma to be transfused should be at least one unit (approximately 200 mL) but may be greater if the treating clinician concludes a larger volume is appropriate. Transfusions may occur at any time throughout the hospitalization including multiple doses on non-sequential days. In general, it is expected that most patients will receive two units or less, but this language is not intended to restrict the use of convalescent plasma in larger quantities when the treating physician determines that such volumes and/or re-treatment are clinically justified.

4.4 CONVALESCENT PLASMA COMPATIBILITY

ABO compatible convalescent plasma will be transfused preferentially. If ABO compatible convalescent plasma is not available, investigators may follow their institution’s guidelines for administration of incompatible plasma with respect to ABO mismatch, titer, and volume limits.

4.5 END OF STUDY DEFINITION

Patients will complete the study when they are discharged from the acute care facility in which they received the COVID-19 convalescent plasma or 30 days after most recent convalescent plasma transfusion whichever comes first.

**5 STUDY POPULATION**

5.1 INCLUSION CRITERIA

1. Age at least 18 years
2. Laboratory confirmed or clinically suspected diagnosis of infection with SARS-CoV-2

Note: Patients enrolled based on clinically suspected infection with SARS-CoV-2 should have pending laboratory confirmation.

1. Admitted to an acute care facility for the treatment of COVID-19 complications
2. Severe or life threatening COVID-19, or judged by the treating provider to be at high risk of progression to severe or life-threatening disease
3. Informed consent provided by the patient or healthcare proxy

Severe COVID-19 is defined by one or more of the following:

- dyspnea
- respiratory frequency ≥ 30/min
- blood oxygen saturation ≤ 93%
- partial pressure of arterial oxygen to fraction of inspired oxygen ratio < 300
- lung infiltrates > 50% within 24 to 48 hours

Life-threatening COVID-19 is defined as one or more of the following:

- respiratory failure
- septic shock
- multiple organ dysfunction or failure

5.2 STRATEGIES FOR RECRUITMENT AND RETENTION

Patients eligible for this Expanded Access Program will be identified by their treating providers and

registered with American Red Cross or other blood source by uscovidplasma.org. Since this involves the administration of COVID-19 convalescent plasma to patients in acute care facilities, participant retention is not anticipated to be an issue.

**6 STUDY INTERVENTION**

6.1 STUDY INTERVENTION(S) ADMINISTRATION

This expanded program will make available compatible COVID-19 convalescent plasma collected by

blood establishments in accordance with the AABB Circular of Information and FDA's regulations and the additional considerations for convalescent plasma on FDA’s webpage ([https://www.fda.gov/vaccines-](https://www.fda.gov/vaccines-blood-biologics/investigational-new-drug-ind-or-device-exemption-ide-process-cber/investigational-covid-19-convalescent-plasma-emergency-inds) [blood-biologics/investigational-new-drug-ind-or-device-exemption-ide-process-cber/investigational-](https://www.fda.gov/vaccines-blood-biologics/investigational-new-drug-ind-or-device-exemption-ide-process-cber/investigational-covid-19-convalescent-plasma-emergency-inds) [covid-19-convalescent-plasma-emergency-inds](https://www.fda.gov/vaccines-blood-biologics/investigational-new-drug-ind-or-device-exemption-ide-process-cber/investigational-covid-19-convalescent-plasma-emergency-inds)).^5^

6.1.1 STUDY INTERVENTION DESCRIPTION

Compatible COVID-19 convalescent plasma will be administered according to standard hospital

procedures.

6.1.2 DOSING AND ADMINISTRATION

For practical purposes in the current outbreak, one – two units of COVID-19 convalescent plasma will be

administered. This will be provided by a registered or licensed blood collector and will be collected preferably by apheresis (total volume of plasma to be administered will be approximately 200 mL or more), or if necessary, by conventional methods (volume of plasma to administer approximately 200- 250 mL per unit). Individual institutional guidelines for the administration of plasma should be followed, including the use of any premedications, such as acetaminophen and diphenhydramine.

**If a subsequent unit of convalescent plasma is transfused**, the treating physician will begin the transfusion at a time that is clinically compatible with the patient’s underlying condition.

6.2 PREPARATION/HANDLING/STORAGE/ACCOUNTABILITY

6.2.1 ACQUISITION AND ACCOUNTABILITY

Convalescent plasma units may be obtained from a registered or licensed blood collector following

registration of the patient.

6.2.2 FORMULATION, APPEARANCE, PACKAGING, AND LABELING

COVID-19 convalescent plasma will be supplied as an investigational blood product for the treatment of

COVID-19.

The container label of COVID-19 convalescent plasma units must include the following statement,

“Caution: New Drug--Limited by Federal (or United States) law to investigational use." (21 CFR 312.6(a)).

In addition, the requirements in 21 CFR 606.121 for the container label applies, including the requirement to include a reference to the circular of information.

6.2.3 PRODUCT STORAGE AND STABILITY

Please see the AABB Circular of Information for product storage and stability.

6.2.4 PREPARATION

Please follow local institutional guidelines for conventional plasma administration for COVID-19 plasma

preparation prior to administration, including compatibility checks and thawing.

6.3 CONCOMITANT THERAPY

Premedications may be administered prior to plasma administration according to individual acute care

facility protocols.

**7 STUDY INTERVENTION DISCONTINUATION AND PARTICIPANT DISCONTINUATION/WITHDRAWAL**

7.1 DISCONTINUATION OF STUDY INTERVENTION

This study involves the administration of COVID-19 convalescent plasma. Patients are free to withdraw

consent from participation at any time.

7.2 PARTICIPANT DISCONTINUATION/WITHDRAWAL FROM THE STUDY

Patients are free to withdraw consent from participation in further data collection at any time during

the study.

Study team encouragement of withdrawal from the EAP as a strategy to avoid completion of follow-up data requirements is not allowed.

7.3 LOST TO FOLLOW-UP

Since this protocol involves only the acute care of individuals with COVID-19, it is not anticipated that

individuals will be lost to follow-up.

**8 STUDY ASSESSMENTS AND PROCEDURES**

8.1 EFFICACY ASSESSMENTS

Patient demographic information will be obtained to include age and sex

Assessments for potential efficacy are exploratory as part of this expanded access program but may include web-based collection of the following information:

1. Acute care facility length of stay
2. Number of days in an intensive care unit
3. Number of days on mechanical ventilation
4. Survival until discharge from an acute care facility

An exploratory analysis may be conducted correlating the level of neutralizing antibody titers with clinical outcomes observed.

8.2 SAFETY AND OTHER ASSESSMENTS

Reporting is required for serious adverse events (SAEs); and we will ask the treating physician when reporting the event to determine if the event is potentially related to the administration of the COVID- 19 convalescent plasma.

8.3 ADVERSE EVENTS AND SERIOUS ADVERSE EVENTS

Serious adverse events should be reported, by the treating physician to the sponsor/principal investigator using the forms provided on the website ([www.uscovidplasma.org](http://www.uscovidplasma.org/)).

The sponsor/principal investigator is responsible for filing reports to the FDA and IRB as required.

8.4 UNANTICIPATED PROBLEMS

Any unanticipated issues should be reported to the principal investigator and to the IND Sponsor,

Michael Joyner by sending an email to [uscovidplasmaevents@mayo.edu.](mailto:uscovidplasmaevents@mayo.edu)

**9 STATISTICAL CONSIDERATIONS**

9.1 STATISTICAL HYPOTHESES

This is an expanded access program and any statistical analyses for safety or efficacy will be exploratory.

9.2 SAMPLE SIZE DETERMINATION

This is an expanded access protocol that is intended to supply COVID-19 convalescent plasma to individuals with severe or life-threatening disease or at high risk thereof. It is anticipated that up to several thousand COVID-19 patients might be enrolled, though this number is difficult to estimate, given the evolving nature of the current pandemic.

9.3 POPULATIONS FOR ANALYSES

Serious adverse event information will be collected on all consented and enrolled patients for whom

convalescent plasma is ordered.

Exploratory analyses will be performed on data obtained from individuals providing informed consent.

9.4 STATISTICAL ANALYSES

Exploratory statistical analyses will be performed as part of this expanded access program.

9.5 PUBLICATION PLAN

The sponsor of this Expanded Access Program will limit the publication of any partial data collected by

any participating treating physician or site without prior written authorization. With rare exceptions, such authorization will likely come after the sponsor has closed program enrollment, completed the data analysis and evaluated the final data set for the primary safety outcomes.

**10 SUPPORTING DOCUMENTATION AND OPERATIONAL CONSIDERATIONS**

10.1 REGULATORY, ETHICAL, AND STUDY OVERSIGHT CONSIDERATIONS

Plasma represents a licensed blood product. However, the use of plasma for the treatment of COVID-19

is investigational.

10.1.1 INFORMED CONSENT PROCESS

Since the use of COVID-19 plasma is investigational at this time, a discussion of potential risks and

benefits to its administration, as well as alternative options, should take place with patients or their healthcare proxies. This should be documented in the medical record.

10.1.1.1 CONSENT/ASSENT AND OTHER INFORMATIONAL DOCUMENTS PROVIDED TO PARTICIPANTS

The IRB approved informed consent form is available on the website [www.uscovidplasma.org](http://www.uscovidplasma.org/). The

language has been deliberately streamlined for use in the setting of Expanded Access in the current COVID-19 pandemic.

10.1.1.2 CONSENT PROCEDURES AND DOCUMENTATION

Consent must be obtained from the patient or the patient's health care proxy prior to treatment.

Expanded access to an investigational drug for treatment use, including emergency use, requires informed consent as described in 21 CFR part 50. Informed consent may be deemed infeasible if, in accordance with 21 CFR 50.23, the investigator and another physician who is not otherwise participating in the protocol certify in writing all of the following:

1. The patient is confronted by a life-threatening situation necessitating use of convalescent plasma
2. Informed consent cannot be obtained from the patient because of an inability to communicate with or obtain legally effective consent
3. Time is not sufficient to obtain consent from the patient’s LAR
4. There is no available alternative method of approved or generally recognized therapy that provides an equal or greater likelihood of saving the life of the patient.

If immediate use of the test article is, in the investigator's opinion, required to preserve the life of the subject, and time is not sufficient to obtain the independent determination required above, the determinations of the investigator shall be made and, within 5 working days after the use of the article, be reviewed and evaluated in writing by a physician who is not participating in the clinical investigation.

The investigator upon documentation of the use of emergency consent notifies the Mayo Clinic IRB via the web based patient entry portal. The study cannot proceed with an order for convalescent plasma until the documentation is complete. The IRB Senior Chair and medical director receives a daily update of study enrollment and key metrics. We will make sure this measure is also included in the daily report.

The computer system will generate a weekly report for the IRB office as a backup of the notification so that the IRB has the original and a backup notification. These will be submitted to the IRB office as notation items.

10.2 IRB RELIANCE

**IRB Reliance**

Your request to participate in the EAP, use of the product and the consent form indicates your willingness and that of your institution/hospital/practice/legal business entity to rely upon the Mayo Clinic IRB and that you will follow all federal and state regulations regarding use and administration of the investigational product and that you will conduct the EAP in accordance with the principles set forth in the Belmont Report.

10.3 SAFETY OVERSIGHT

## Safety Oversight

The Mayo Clinic IRB, a DSMB and the US FDA will work collaboratively to follow SAE reporting and monitor the conduct of the EAP.

The DSMB will be composed of experienced physicians and scientists who understand the risks of administered products and have sufficient research and trial experience to provide an independent recommendation to the PI, the IRB and the FDA. The FDA will hold the ultimate decision-making power to terminate the study evidence of early benefit or harm. The DSMB will generate regular reports to the IRB and FDA., The DSMB Chair, the regulatory sponsor, and additional team memebers will communicate with the appropriate FDA officials for review and guidance on study conduct. IND Safety Reports will be submitted to the FDA as required under the IND.

DSMB Membership

The DSMB membership will include:

Allan S. Jaffe, MD, - Chair

Professor of Medicine and Laboratory Medicine

Consultant in the Division of Cardiac Critical Care and Ischemic Heart Disease Mayo Clinic, Rochester, MN

David O. Warner, MD - Secretary Professor ofAnesthesiology Mayo Clinic, Rochester, MN

William Morice, MD

Professor of Laboratory Medicine

Chair, Department of Laboratory Medicine and Pathology Mayo Clinic, Rochester, MN

Paula Santrach, MD

Associate Professor of Laboratory Medicine and Pathology Consultant in Transfusion Medicine

Mayo Clinic, Rochester, MN

Robert L. Frye, MD

Professor of Medicine, Mayo Clinic and Past Chair, Department of Medicine Mayo Clinic, Rochester, MN

Lawrence J. Appel, MD, MPHC. David Molina, M.D., M.P.H. Professor of Medicine and International Health, and Nursing

Director, Welch Center for Prevention, Epidemiology, and Clinical Research Johns Hopkins Medical Institution

Rickey E. Carter Ph.D. Professor of Biostatistics Mayo Clinic, Jacksonville Fl.

Ex Officio

Taimur Sher, MD, Associate Professor of Medicine, Co-Chair Mayo Clinic Thursday IRB

Consultant in the Division of Hematology Mayo Clinic, Jacksonville, FL

Kristine Tree

Center for Individualized Medicine Mayo Clinic, Rochester, MN

10.4 ABBREVIATIONS

| AE | Adverse Event |
| --- | --- |
| CFR | Code of Federal Regulations |
| CRF | Case Report Form |
| EC | Ethics Committee |
| eCRF | Electronic Case Report Forms |
| FDA | Food and Drug Administration |
| IB | Investigator’s Brochure |
| IND | Investigational New Drug Application |
| IRB | Institutional Review Board |
| NIH | National Institutes of Health |
| SAE | Serious Adverse Event |
| SAP | Statistical Analysis Plan |
| SOP | Standard Operating Procedure |
| US | United States |

10.5 PROTOCOL AMENDMENT HISTORY

| **Version** | **Date** | **Description of Change** | **Brief Rationale** |
| --- | --- | --- | --- |
| 1.0 | 4/1/2020 | Initial Version | N/A |
| 2.0 | 4/3/2020 | Updated IND Number Added IRB Oversight | Documentation Clarification |
| 3.0 | 4/9/2020 | Updated Section 10.1.1.2  Consent Procedures and Documentation | Additional consenting procedures for patients unable to give consent and also no LAR available to give consent for patients qualifying for plasma infusion. |
| 4.0 | 4/15/2020 | Updated Section 6.1.2 | Clarify 1-2 units of plasma and up to 500ml of volume delivered |
| 5.0 | 4/23/3030 | Study synopsis clarified to be consistent throughout 1-2 units  Section 1.2 and 5.2 Eligible patients will be registered with American Red Cross or other blood source by uscovidplasma.org  Section 1.2 Clarify infusion rate for plasma deleted specific rate.  Updated Sections 4.3 and 6.1.2 to define if 2 units are transfused the second unit must be transfused within 12 hours of first.  Updated Labeling in Section 6.2.2  Section 8.2. and 8.3 SAE reporting should be done using forms on website.  Section 9.3 Clarified collection of event information  Added Section 9.5 for Publication plans  10.1.1.1 Revised reference to informed consent document  Section 10.3 Clarified safety monitoring and updated DSMB Roster | Consistency throughout protocol that 1-2 units of plasma may be administered  Clarification and consistency to coordinate patients and available plasma  Allow for flexibility to individual institutional and patient standards for care  Reduce deviations and allow flexibility in scheduling if 2 units are administered.  Align with current FDA Guidance. Clarify SAE reporting  Information should be collected from all consented and enrolled patients for whom plasma is ordered  Clarify Publication Plan  IRB Approved Consent is available on the website not part of this document.  Clarification and updates |
| 6.0 | 5/15/2020 | Throughout the protocol, removed reference to ABO  Section 4.3 - Dose justification of volume of convalescent plasma | See New Section 4.4  The volume of plasma to be transfused should be based upon the patient’s weight and clinical comorbidities and allowance for multiple doses. |

| **Version** | **Date** | **Description of Change** | **Brief Rationale** |
| --- | --- | --- | --- |
|  |  | Section 4.4 - Added to define preference for ABO compatible.  Sections 5.2 & 7.1 - Clarifications for dosing and adminstration  Section 6.1.2 - Dosing and administration changed to subsequent units  Section 10.1.1.2 - Process for informing the IRB of the number of Emergency Use consent clarified. | ABO compatible convalescent plasma will be transfused preferentially. In the absence of ABO compatible plasma, patients may receive as a second choice either Group A plasma or low anti-A titer Group O plasma, as available  To provide consistency between sections 4.3, 5.2, and 7.1.  To align with Section 4.3  To maintain communication with the IRB to provide the number of cases of Emergency Use consenting. |
| 7.0 | 5/23/2020 | Throughout the protocol, changed the description of the investigational product to just be Convalescent Plasma.  Section 4.4 - Changed the description for plasma compatibility, ABO compatible convalescent plasma will be transfused preferentially. If ABO compatible convalescent plasma is not available,  investigators may follow their institution’s guidelines for administration of incompatible plasma with respect to ABO mismatch, titer, and volume limits | The term “compatible” was removed since there is now the alternative to follow their  institution’s guidelines for administration of incompatible plasma with respect to ABO mismatch, titer, and volume limits  FDA Request for clarifications |
| 8.0 | 6/16/2020 | Section 4.5 - End of study definition is either discharge from acute care facility, or, 30 day after most recent COVID-19 convalescent plasma transfusion.  Section 6.2.2 - Labeling of COVID-19 convalescent plasma should include a label to indicate presence of COVID-19 antibodies, if testing is available.  Section 8.4 – Added specific email address for reporting of other unanticipated issues. [uscovidplasmaevents@mayo.edu](mailto:uscovidplasmaevents@mayo.edu) | Clarification of end of study definition.  Alignment with guidance from FDA.  Specially designated email for reporting other unanticipated issues. |
| 9.0 | 7/15/2020 | Section 1.2 and 5.1 Eligibility criteria - added clinically suspected along with laboratory confirmed diagnosis.  Section 6.2.2 – Removed Labeling of COVID-19 convalescent plasma should include a label to indicate presence of COVID-19 antibodies, if testing is available. | Allow participation of patients with either laboratory confirmed or clinically suspected SARS-CoV-2 to be eligible.  Alignment with more recent (5/1/2020) [*FDA*](https://www.fda.gov/media/136798/download) [*Guidance for Industry - Investigational*](https://www.fda.gov/media/136798/download) [*COVID-19 Convalescent Plasma*.](https://www.fda.gov/media/136798/download) |

| **Version** | **Date** | **Description of Change** | **Brief Rationale** |
| --- | --- | --- | --- |
| 10.0 | 8/3/2020 | Section 1.2 and 5.1 Eligibility criteria suspected diagnosis should include a pending laboratory test result. | Any patient enrolled based on clinically suspected infection with SARS-CoV-2 should have pending laboratory confirmation.  Recommendation from FDA 7/31/2020. |

**11 REFERENCES**

REFERENCES

1. Roback JD, Guarner J, Convalescent Plasma to Treat COVID-19: Possibilities and Challenges., JAMA. 2020 Mar 27;

[Pmid:32219429](https://www.ncbi.nlm.nih.gov/PubMed/32219429)

1. Shen C, Wang Z, Zhao F, Yang Y, Li J, Yuan J, Wang F, Li D, Yang M, Xing L, Wei J, Xiao H, Yang Y, Qu J, Qing L, Chen L, Xu Z, Peng L, Li Y, Zheng H, Chen F, Huang K, Jiang Y, Liu D, Zhang Z, Liu Y, Liu L, Treatment of 5 Critically Ill Patients With COVID-19 With Convalescent Plasma., JAMA. 2020 Mar 27;

[Pmid:32219428](https://www.ncbi.nlm.nih.gov/PubMed/32219428)

1. Mair-Jenkins J, Saavedra-Campos M, Baillie JK, Cleary P, Khaw FM, Lim WS, Makki S, Rooney KD, Nguyen- Van-Tam JS, Beck CR, Convalescent Plasma Study Group., The effectiveness of convalescent plasma and hyperimmune immunoglobulin for the treatment of severe acute respiratory infections of viral etiology: a systematic review and exploratory meta-analysis., J Infect Dis. 2015 Jan 1;211(1):80-90

[Pmid:25030060](https://www.ncbi.nlm.nih.gov/PubMed/25030060)

1. Ko JH, Seok H, Cho SY, Ha YE, Baek JY, Kim SH, Kim YJ, Park JK, Chung CR, Kang ES, Cho D, Müller MA, Drosten C, Kang CI, Chung DR, Song JH, Peck KR, Challenges of convalescent plasma infusion therapy in Middle East respiratory coronavirus infection: a single centre experience., Antivir Ther. 2018;23(7):617- 622

[Pmid:29923831](https://www.ncbi.nlm.nih.gov/PubMed/29923831)

1. <http://www.aabb.org/tm/coi/Pages/default.aspx>

**Study Protocol Amendments Summary**

| **Version** | **Date** | **Description of Change** | **Brief Rationale** |
| --- | --- | --- | --- |
| 1.0 | 4/1/2020 | Initial Version | N/A |
| 2.0 | 4/3/2020 | Updated IND Number Added IRB Oversight | Documentation Clarification |
| 3.0 | 4/9/2020 | Updated Section 10.1.1.2  Consent Procedures and Documentation | Additional consenting procedures for patients unable to give consent and also no LAR available to give consent for patients qualifying for plasma infusion. |
| 4.0 | 4/15/2020 | Updated Section 6.1.2 | Clarify 1-2 units of plasma and up to 500ml of volume delivered |
| 5.0 | 4/23/3030 | Study synopsis clarified to be consistent throughout 1-2 units  Section 1.2 and 5.2 Eligible patients will be registered with American Red Cross or other blood source by uscovidplasma.org  Section 1.2 Clarify infusion rate for plasma deleted specific rate.  Updated Sections 4.3 and 6.1.2 to define if 2 units are transfused the second unit must be transfused within 12 hours of first.  Updated Labeling in Section 6.2.2  Section 8.2. and 8.3 SAE reporting should be done using forms on website.  Section 9.3 Clarified collection of event information  Added Section 9.5 for Publication plans  10.1.1.1 Revised reference to informed consent document  Section 10.3 Clarified safety monitoring and updated DSMB Roster | Consistency throughout protocol that 1-2 units of plasma may be administered  Clarification and consistency to coordinate patients and available plasma  Allow for flexibility to individual institutional and patient standards for care  Reduce deviations and allow flexibility in scheduling if 2 units are administered.  Align with current FDA Guidance. Clarify SAE reporting  Information should be collected from all consented and enrolled patients for whom plasma is ordered  Clarify Publication Plan  IRB Approved Consent is available on the website not part of this document.  Clarification and updates |
| 6.0 | 5/15/2020 | Throughout the protocol, removed reference to ABO  Section 4.3 - Dose justification of volume of convalescent plasma | See New Section 4.4  The volume of plasma to be transfused should be based upon the patient’s weight and clinical comorbidities and allowance for multiple doses. |

| **Version** | **Date** | **Description of Change** | **Brief Rationale** |
| --- | --- | --- | --- |
| 6.0 | 5/15/2020 | Section 4.4 - Added to define preference for ABO compatible.  Sections 5.2 & 7.1 - Clarifications for dosing and adminstration  Section 6.1.2 - Dosing and administration changed to subsequent units  Section 10.1.1.2 - Process for informing the IRB of the number of Emergency Use consent clarified. | ABO compatible convalescent plasma will be transfused preferentially. In the absence of ABO compatible plasma, patients may receive as a second choice either Group A plasma or low anti-A titer Group O plasma, as available  To provide consistency between sections 4.3, 5.2, and 7.1.  To align with Section 4.3  To maintain communication with the IRB to provide the number of cases of Emergency Use consenting. |
| 7.0 | 5/23/2020 | Throughout the protocol, changed the description of the investigational product to just be Convalescent Plasma.  Section 4.4 - Changed the description for plasma compatibility, ABO compatible convalescent plasma will be transfused preferentially. If ABO compatible convalescent plasma is not available,  investigators may follow their institution’s guidelines for administration of incompatible plasma with respect to ABO mismatch, titer, and volume limits | The term “compatible” was removed since there is now the alternative to follow their  institution’s guidelines for administration of incompatible plasma with respect to ABO mismatch, titer, and volume limits  FDA Request for clarifications |
| 8.0 | 6/16/2020 | Section 4.5 - End of study definition is either discharge from acute care facility, or, 30 day after most recent COVID-19 convalescent plasma transfusion.  Section 6.2.2 - Labeling of COVID-19 convalescent plasma should include a label to indicate presence of COVID-19 antibodies, if testing is available.  Section 8.4 – Added specific email address for reporting of other unanticipated issues. [uscovidplasmaevents@mayo.edu](mailto:uscovidplasmaevents@mayo.edu) | Clarification of end of study definition.  Alignment with guidance from FDA.  Specially designated email for reporting other unanticipated issues. |
| 9.0 | 7/15/2020 | Section 1.2 and 5.1 Eligibility criteria - added clinically suspected along with laboratory confirmed diagnosis.  Section 6.2.2 – Removed Labeling of COVID-19 convalescent plasma should include a label to indicate presence of COVID-19 antibodies, if testing is available. | Allow participation of patients with either laboratory confirmed or clinically suspected SARS-CoV-2 to be eligible.  Alignment with more recent (5/1/2020) [*FDA*](https://www.fda.gov/media/136798/download) [*Guidance for Industry - Investigational*](https://www.fda.gov/media/136798/download) [*COVID-19 Convalescent Plasma*.](https://www.fda.gov/media/136798/download) |
| **Version** | **Date** | **Description of Change** | **Brief Rationale** |
| 10.0 | 8/3/2020 | Section 1.2 and 5.1 Eligibility criteria suspected diagnosis should include a pending laboratory test result. | Any patient enrolled based on clinically suspected infection with SARS-CoV-2 should have pending laboratory confirmation.  Recommendation from FDA 7/31/2020. |

**Statistical Analysis Plan (version 2.0)**

**Data set description:** As of **5/28/2020**, there are 17,155 transfusions. We estimate approximately 15,000 of these will have data through Day 7 by the end of next week. Outcome data through Day 30 will be available on a much smaller set of patients, and given the interval censoring between Day 7 and Day 30 with the current data structure, analyses for this analysis report will focus on all-cause mortality through Day 7.

**Primary Hypothesis:** Antibody concentration level in transfused plasma will establish a dose-response relationship with overall survival in patients with COVID-19 treated with convalescent plasma.

**Secondary Hypothesis:** The effect of antibody concentration level will serve as an effect modifier in the presence of baseline measures of disease severity and pre-disposing risk factors.

**Primary Analysis Plan**

Preparation of the survival endpoint: The timing of death is recorded only with the precision of a calendar day, so adjustments are needed to develop the survival estimates. For deaths that occur on the same day of transfusion, a death indicator representing 0.5 days will be administratively assigned. For deaths out to day 7, integer counts of the number of calendar days post transfusion will be computed. For records of patients transfused but do not contain any post transfusion data, these patients will be administratively censored at day 0.25. Otherwise, the length of known vital status will be used to determine inclusion in the risk set (e.g., reported alive in the daily summary, assessed for the day 7 follow up, etc).

The primary analysis of antibody titer data will first consider a Cox proportional hazards model using a spline fit on the observed antibody concentration line. The spline is to avoid a strong linear assumption on the relationship of the titer and survival. At least three knots will be considered to allow the dose-response relationship to follow a general sigmoid shape if one is present in the data. A partial likelihood test will be constructed to test for the main effect of antibody levels in the data. Should this test be statistically significant at the alpha=0.05 level of significance (two-sided), simplifications of the model to facilitate secondary analyses will be constructed. Using partial dependence plots, the general functional form of the association of antibody concentration will be associated with the probability for mortality. Without loss of generality, we assume there will be low, moderate and high concentration groups that will be identified (i.e., the three main pieces of the hypothesized sigmoid dose-response relationship). Using these as defined strata, a log rank test will be constructed using these categories.

Secondary data analyses to assess for risk modification can follow in two ways. First variable by concentration interactions terms can be added to the Cox model. While this approach is statistically efficient, the complexity of the resulting model will make real-world interpretations of the findings challenging. To address this, a Breslow-Day stratified analysis approach will be considered.

For this analysis, 7 day estimated mortality rates will be estimated using the product limit estimator for each stratum of the data. This approach will follow a generally hierarchical process. First, as noted above, the data will be stratified by antibody concentration level (e.g., 3 strata for low, moderate and high concentrations). Next, stratification will be based on admission to ICU at time of transfusion, yes or no, which result in 6 strata (e.g., 3x2). This general approach will continue with additional risk modifiers as indicated in the appendix to result in finer and finer sub categorization of patients. These estimates will be visualized with forest plots to provide an overall impression of heterogeneity among the risk modifiers. As with the primary analysis, the data will be grouped so that the three antibody concentrations levels will be collocated on the figure to provide some evidence of the dose-response relationship, if one exists. Formal tests for the effect modification will be conducted using the Cox framework using appropriately chosen interaction terms.

**Risk Modifiers**

1. COVID-19 Severity
2. At risk of severe / life threatening COVID-19
3. Currently has severe / life threatening COVID-19 with further delineation of why (Y/N to each of the following: dyspnea, respiratory rate >=30/min, spo2 <= 93%, p/f <300, lung infiltrates > 50 within 24 – 48 hrs, respiratory failure, septic shock, multi-organ dysfunction or failure)
4. Body Composition: Height, Weight, BMI
5. Race
6. Ethnicity
7. Gender/Sex
8. No. of days from Covid-19 symptom onset to convalescent plasma transfusion
9. No. of hospitalized days prior to convalescent plasma transfusion
10. No. of days in the ICU prior to convalescent plasma transfusion
11. No. of days receiving mechanical ventilation prior to convalescent plasma transfusion
12. Pre-existing conditions
    1. Lung disease
    2. Cardiovascular conditions
    3. Obesity
    4. HIV
    5. HCV
    6. Immunosuppressive therapy
    7. Diabetes
13. Prior Medications
    1. ACEI
    2. ARBs
14. Medications during Hospitalization
    1. ACEI
    2. ARBs
    3. Azithromycin
    4. Remdesivir
    5. Steroids
    6. Chloroquine
    7. Hydroxycloroquine
15. Highest level of respiratory support prior to convalescent plasma transfusion
    1. Non-invasive positive pressure
    2. Mechanical ventilation
    3. ECMO

**Statistical Analysis Plan (version 5.0)**

**Date:** 27 July 2020

## Statistical Hypotheses

**Primary Hypothesis:** 7-Day and 30-Day mortality will be affected by plasma transfusion dynamics after adjustment for pre-disposing risk factors. Transfusion dynamics are defined in the context of timeliness of transfusion relative to date of diagnosis, the volume of plasma initially given (i.e., on the first transfusion day), and the quantity of neutralizing antibodies on the first day of transfusion.

## Primary Analysis Plan

### Preparation of the survival endpoint: The timing of death is recorded only with the precision of a calendar day, so adjustments are needed to develop the survival estimates. For deaths that occur on the same day of transfusion, a death indicator representing 0.5 days will be administratively assigned. Otherwise, the number of days between the date of the first transfusion and death will be calculated for each participant. For patients transfused without a death record recorded as an SAE, or more precisely, on the death case report form, they are assumed to be alive. Within the context of the report and programming, this is denoted as the "immortal" survival assumption. To minimize extrapolation of the survival curves beyond a reasonable time that this assumption could be upheld, survival estimates are limited to 30 days; however, the report does introduce stopping the survival curves early, for example, at Day 7 for 7-Day mortality estimates.

### Primary Data Analysis: The primary analysis will be conducted through a series of unadjusted and adjusted survival models. This is required given the study is non-randomized and factors that may affect overall survival may serve as a confounding variable in the analysis. The primary hypothesis presents three variables that are hypothesized to have an impact on mortality.

First, the time to transfusion is considered. Based on early reports with convalescent plasma, a target window of 0 days, 1-3 days was considered optimal. Therefore, patients are divided into a group representing "timely" delivery of convalescent plasma. The remaining subjects are split into two groups, Days 4-10 (i.e., a week following a timely period) and Days 11+. In some analyses to aide in the graphical presentation of the data, the timeliness data is dichotomized into <=3 days vs. 4+ days.

Second, the transfusion volume on the first day of transfusion (if more than 1 transfusion was given). For this analysis, various categorizations of the volumes were considered, however, for the primary analysis, a spilt at 225 ml was implemented. This effectively separates people that received 1 unit (~ 200ml) from those that deliberately received more than 1 unit of plasma. 225 ml was selected to all for some variance in the volume in the first unit of plasma.

For the final component of the analysis, the neutralizing antibodies are both semi-quantitative and categorical in interpretation. There are lab established thresholds for negative (<1:80) and serial dilution allows for estimates ranging from 1:80 to >=1:2560. The analysis will consider the levels of the titers as nominal values as well as introducing some thresholds to classify the titers into a "low" vs. "high" classification. The original statistical analysis plan consisted of a planned analysis of the titer data using a splined fit on the observed antibody concentration. The spline was to avoid a strong linear assumption on the relationship of the titer and survival. The decision to use that approach was changed post hoc to the dichotomized approaches to account for the lack of dose-response relationship observed with the data. This decision allowed for simplification of the data presentation.

Crude mortality and 95% (score) confidence intervals (CI) are calculated for the overall cohort and additionally, by some of the key variables of interest. Given the study is non-randomized and multiple sources of confounding are potential present in the data, adjusted estimates are also provided in the report. The approach for adjusted estimate is as follows:

- A baseline Cox regression model is fit to the data. Without loss of generality, assume there is one variable of direct interest (e.g., volume of plasma transfusion) and a set of covariates that are desired to be controlled for in the estimate.
- Using the 'conditional' method for estimating adjusted survival curves (https://cran.r-project.org/web/packages/survival/vignettes/adjcurve.pdf), an adjusted estimate of the mortality is obtained. This process allows for estimation of an expected survival at a particular point in time (e.g., at 30 days).
- To estimate the confidence interval for the adjusted survival curve, the bootstrap is used. For each of the replicates performed, the original data object is used to determine the reference distribution for standardized mortality estimates.
- The approach was extended to provide an estimate of the relative risk over one or more variables of interest. A posterior distribution of potential relative risks was constructed by a cartesian merge of the posterior adjusted survival estimates for each group. The 2.5th and 97.5th percentiles of this distribution were used as the bootstrap confidence interval for the relative risk. No p-values are provided for this method. In events where statistical inference is desired, the hazard ratio and associated significance test should be utilized.

### Secondary Data Analysis: In the future, additional secondary data analyses to assess for risk modification can follow in two ways. First variable by concentration interactions terms can be added to the Cox model. While this approach is statistically efficient, the complexity of the resulting model will make real-world interpretations of the findings challenging. To address this, a Breslow-Day stratified analysis approach will be considered. For this initial report, separate results by strata of interest are presented. The strata presented represent important subsets of the data that warrant further consideration.

For this future analysis, estimated mortality rates will be estimated using the product limit estimator for each stratum of the data. This approach will follow a generally hierarchical process. First, as noted above, the data will be stratified by time of transfusion, infusion volume, and antibody concentration level. Next, stratification will be based on admission to ICU at time of transfusion, yes or no, which result in multiple strata of interest (e.g., ICU patients transfused within 3 days using 2 units of plasma with high concentration of neutralizing antibodies). This general approach will continue with additional risk modifiers as indicated below to result in finer and finer sub categorization of patients. These estimates will be visualized with forest plots to provide an overall impression of heterogeneity among the risk modifiers. As with the primary analysis, the data will be grouped so that the three antibody concentrations levels will be collocated on the figure to provide some evidence of the dose-response relationship, if one exists. Formal tests for the effect modification will be conducted using the Cox framework using appropriately chosen interaction terms.

**Risk Modifiers**

1. Calendar month of Covid-19 convalescent plasma transfusion
2. Age
3. Gender/Sex
4. Race
5. Ethnicity
6. BMI
7. ICU at time of Covid-19 convalescent plasma transfusion
8. Level of respiratory support at time of Covid-19 convalescent plasma transfusion
9. Body Composition: Height, Weight, BMI
10. No. of days from Covid-19 symptom onset to convalescent plasma transfusion
11. COVID-19 Severity
12. At risk of severe / life threatening COVID-19
13. Currently has severe / life threatening COVID-19 with further delineation of why (Y/N to each of the following: dyspnea, respiratory rate >=30/min, spo2 <= 93%, p/f <300, lung infiltrates > 50 within 24 – 48 hrs, respiratory failure, septic shock, multi-organ dysfunction or failure)

**Study Protocol Amendments Summary**

| **Version** | **Description of Change** |
| --- | --- |
| **1.0** | Not applicable. |
| **2.0** | The initial statistical vignette was developed into a robust statistical analysis plan with consideration of putative risk factors identified in contemporary Covid-19 clinical trials. |
| **3.0** | Additional details regarding the performance characteristics of a semiquantitative, pseudo neutralizing antibody assay revealed a range of six categorical dilution coefficients (1:80, 1:160, 1:320, 1:640, 1:1280, 1:2560), that informed the stratification of anti-SARS-CoV-2 antibody level as “low”, “medium” and “high”. |
| **4.0** | An additional component of the analysis, considering the effects of timeliness of transfusion and categorical antibody level was added. Six stratifications (3×2) were planned, using a dichotomous split for timeliness (with 3 days vs. 4 or more days) and three anti-SARS-CoV-2 antibody levels. |
| **5.0** | In an extension of the baseline Cox regression model fitted to the data, a combination of ‘conditional’ method for estimating survival and bootstrap methods were used to estimate the relative risk of mortality. |
| **6.0** | An exploratory analysis using a machine learning approach to link mortality with both anti-SARS-CoV-2 antibody levels and adjustment variables was developed during the drafting of a previous manuscript. |

1. Study Protocol Version 1.0 was a draft. Study Protocol Version 2.0 represents the initial protocol document approved by the Mayo Clinic Institutional Review Board (IRB) on April 4, 2020. [↑](#footnote-ref-1)
2. Study Protocol Amendment history is embedded in each Study Protocol Version, and has been duplicated in a separate section for clarity. [↑](#footnote-ref-2)
3. Statistical Analysis Plan 1.0 was a vignette describe in Section 8 of the Study Protocol. Statistical Analysis Plan 2.0 represents the initial descriptive analysis plan. [↑](#footnote-ref-3)
